# CDK4/6 Inhibitors Impede Chemoresistance and Inhibit Tumor Growth of Small Cell Lung Cancer

**DOI:** 10.1002/advs.202400666

**Published:** 2024-08-13

**Authors:** Yang Wen, Xue Sun, Lingge Zeng, Shumei Liang, Deyu Li, Xiangtian Chen, Fanrui Zeng, Chao Zhang, Qiongyao Wang, Qinsong Zhong, Ling Deng, Linlang Guo

**Affiliations:** ^1^ Department of Pathology Zhujiang Hospital Southern Medical University Guangzhou 510080 China; ^2^ Department of Pathology Guangzhou First People's Hospital School of Medicine South China University of Technology Guangzhou 510180 China; ^3^ Department of Pathology Guangzhou First People's Hospital Guangzhou Medical University Guangzhou 510180 China; ^4^ Department of Oncology Fujian Provincial Hospital Fuzhou 350001 China; ^5^ Department of Radiation Oncology The First Affiliated Hospital of Guangzhou Medical University Guangzhou 510120 China; ^6^ Department of Oncology Zhujiang Hospital Southern Medical University Guangzhou 510080 China

**Keywords:** AMBRA1, autophagic flux, CDK6, chemoresistance, lysosomal depletion, SCLC

## Abstract

Small cell lung cancer (SCLC) is characterized by rapid development of chemoresistance and poor outcomes. Cyclin‐dependent kinase 4/6 inhibitors (CDK4/6is) are widely used in breast cancer and other cancer types. However, the molecular mechanisms of CDK4/6 in SCLC chemoresistance remain poorly understood. Here, Rb1^flox/flox^, Trp53^flox/flox^, Pten^flox/flox^ (RTP) and Rb1^flox/flox^, Trp53^flox/flox^, Myc^LSL/LSL^ (RPM) spontaneous SCLC mouse models, SCLC cell line‐derived xenograft (CDX) models, and SCLC patient‐derived xenograft (PDX) models are established to reveal the potential effects of CDK4/6is on SCLC chemoresistance. In this study, it is found that CDK4/6is palbociclib (PD) or ribociclib (LEE) combined with chemotherapeutic drugs significantly inhibit SCLC tumor growth. Mechanistically, CDK4/6is do not function through the classic Retionblastoma1 (RB) dependent axis in SCLC. CDK4/6is induce impair autophagy through the AMBRA1‐lysosome signaling pathway. The upregulated AMBRA1 protein expression leads to CDK6 degradation via autophagy,  and the following TFEB and TFE3 nuclear translocation inhibition leading to the lysosome‐related genes levels downregulation. Moreover, it is found that the expression of CDK6 is higher in SCLC tumors than in normal tissue and it is associated with the survival and prognosis of SCLC patients. Finally, these findings demonstrate that combining CDK4/6is with chemotherapy treatment may serve as a potential therapeutic option for SCLC patients.

## Graphical abstract

In this study, CDK4/6is prevent the growth and chemoresistance formation of Small cell lung cancer (SCLC) via AMBRA1‐CDK6‐TFEB/TFE3 signal pathway.  CDK6 is the major upregulated factor during the resistance period of SCLC chemotherapy, and CDK4/6 inhibitors induce impaired autophagy and cell apoptosis by regulating lysosome function. Collectively, these data identify CDK4/6 inhibitors as a potential therapy for SCLC chemoresistance.

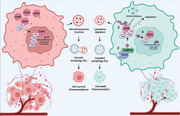

## 1 Introduction

SCLC is an aggressive disease that accounts for ≈15% of lung cancer cases.^[^ ^1^ ^]^ SCLC is a malignancy with a poor prognosis and is characterized by rapid growth, early metastatic spread, and initial responsiveness to therapy.^[^ ^2^ ^]^ The mainstay first‐line treatment for metastatic SCLC is chemotherapy, generally a platinum and etoposide or irinotecan combination. For nonmetastatic disease, prophylactic cranial irradiation should be considered for patients without metastases after induction chemotherapy and radiotherapy.^[^ ^3^ ^]^ SCLC is remarkably sensitive to first‐line platinum chemotherapy, with over 50% of patients responding. However, after the transient impressive responses, the median progression‐free survival in clinical trials is less than 5 months.^[^ ^4^ ^]^ The underlaying molecular mechanisms responsible for this shift between the first impressive responses and later chemoresistance in SCLC need to be identified.^[^ ^5^ ^]^ As a neuroendocrine programmed tumor, SCLC is pathologically and molecularly different from the other lung cancer types. Inactivation of *TP53* and *RB1* are common genetic alterations in SCLC.^[^ ^6^ ^]^ Other signaling pathways are frequently interrupted such as Notch signaling and nuclear factor kappa light chain enhancer of activated B cells signaling.^[^ ^4^, ^7^ ^]^ The high mutational burden of SCLC might provide opportunities for the therapeutic intervention.^[^ ^1a^ ^]^

Pharmacologic inhibitors of CDK4/6 have shown promising effects in patients with breast and other cancer types.^[^ ^8^ ^]^ The dominant mechanisms of CDK4/6 inhibitors are inhibition of RB1 protein phosphorylation and induction of cell cycle arrest.^[^ ^9^ ^]^ Recent studies have suggested that CDK4/6 inhibitors alter cancer cell biology in other ways, such as by regulating the modulation of mitogenic kinase signaling, enhancing cancer cell immunogenicity and inducing an aging phenotype in cancer cells.^[^ ^10^ ^]^ However, almost all signals promote cell cycle progression by mediating the function of RB1 via mono‐ and subsequent hyperphosphorylation Cyclin‐CDK complexes.^[^ ^11^ ^]^ The new generation of selective CDK4/6 inhibitors, including palbociclib, ribociclib and abemaciclib, are most effective in combination with endocrine therapy in patients with breast cancer and other cancer types.^[^ ^12^ ^]^ Recently, a study showed that CDK4/6 inhibition enhanced chemotherapy efficacy by enhancing T‐cell activation in patients with SCLC receiving chemotherapy.^[^ ^13^ ^]^ However, a clinical study indicated that administration of the CDK4/6 inhibitor tranaciclib before chemotherapy improved patient health‐related quality of life, with no impact on chemotherapy treatment of extensive‐stage small cell lung cancer.^[^ ^14^ ^]^ In addition to inducing cell cycle arrest, other mechanisms by which CDK4/6 inhibitors exert antitumor activity need to be clarified.

The latest studies have suggested that autophagy has a critical role in the chemotherapy resistance of SCLC cells. Some studies have shown that autophagic flux is improved during the period of chemotherapy resistance and that impaired autophagic flux can finally trigger apoptosis of SCLC cells.^[^ ^15^ ^]^ In multicellular organisms, formed autophagosomes undergo a process called “maturation”. In this “maturation” stage, they fuse with vesicles originating from endolysosomal compartments, such as early and late endosomes and lysosomes. Impaired autophagosome fusion with lysosomes is linked to the pathogenesis of various human diseases, including neurodegenerative disorders, kidney disease and cancer.^[^ ^16^ ^]^ Recent progress in autophagic flux regulation has indicated that impaired autophagic flux may be a potential therapeutic target to combat SCLC chemoresistance. Furthermore, another study showed that AMBRA1are the main regulators of Cyclin D1 degradation. These data reveal a close relationship between cell cycle kinase and autophagy regulation and provide new insight into the treatment of small cell lung cancer with chemotherapy resistance.^[^ ^17^ ^]^

In this study, we show the potential effect of CDK4/6 inhibitors (palbociclib and ribociclib) on SCLC chemoresistance and tumor growth through SCLC mouse models and SCLC PDX models. Moreover, we found that CDK6 is the major upregulated factor during the resistance period of SCLC chemotherapy, and CDK4/6 inhibitors significantly abolished CDK6 protein upregulation in SCLC chemotherapy‐resistant cells (H69AR & H446DDP). Mechanistically, CDK4/6 inhibitors induce impaired autophagy and cell apoptosis by regulating AMBRA1‐CDK6‐TFE3/TFEB‐lysosome function. Collectively, these data reveal a relationship between CDK6 and autophagic flux regulation and identify CDK4/6 inhibitors as a potential therapy for SCLC chemoresistance.

## 2 Results

### 2.1 Cyclin‐Dependent Kinase 6 Is Involved in SCLC Chemoresistance

**Figure 1:**
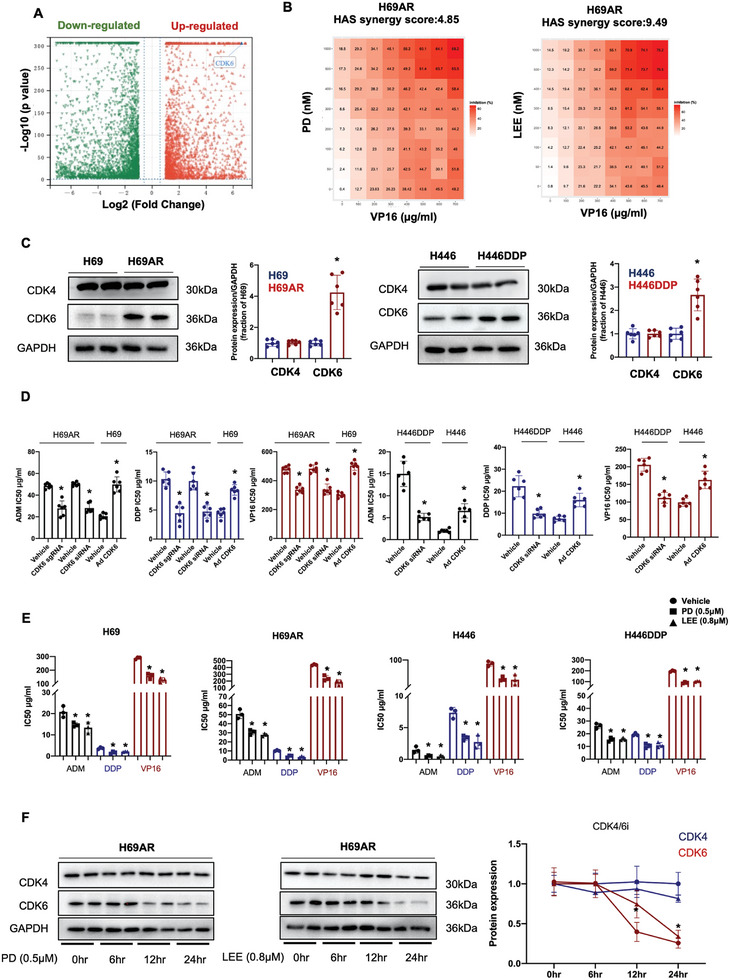
Cyclin‐dependent kinase 6 is involved in chemoresistance of SCLC. A) Differentially expressed genes in H69AR cells compared with H69 cells determined by RNA sequencing, *p* < 0.05. B) The synergy score of PD and LEE with VP16, etoposide of cell viability inhibiton in H69AR cells. C) The protein expression of CDK4 and CDK6 in H69, H69AR, H446, and H446DDP cells (*n* = 6). D) IC50 values detected in CDK6 sgRNA‐transfected H69AR cells, CDK6 siRNA‐transfected H69AR cells, CDK6‐overexpressing adenovirus‐infected H69 cells, CDK6 siRNA‐transfected H446DDP cells, and CDK6‐overexpressing adenovirus‐infected H446 cells treated with or without chemotherapeutic drugs. ADM, adriamycin; DDP, cisplatin; VP16, etoposide. E) IC50 values detected in H69, H69AR, H446, and H446DDP cells treated with or without PD (0.5 µm) and LEE (0.8 µm). PD, palbociclib; LEE, ribociclib. (*n* = 6). F) CDK4 and CDK6 protein expression in H69AR cells treated with PD (0.5 µm) and LEE (0.8 µm) for 0, 6, 12, or 24 h. The right panel is the statistical graph of CDK4 and CDK6 protein expressions. The data are shown as the mean ± SD, *p < 0.05.

To assess the gene expression profiles of chemoresistant SCLC cells, we first performed mRNA sequencing and identified 5374 differentially expressed genes. A total of 1307 genes exhibited upregulated expression and 3067 exhibited downregulated expression in H69AR cells compared with H69 cells (**Figure** **1**A). CDK6 was one of the genes upregulated in H69AR cells compared with H69 cells, and we verified some of the upregulated mRNA results via qPCR and western blotting. CDK4 is a protein closely related to CDK6 and is critical in cell cycle regulation. We performed qPCR analysis and found that CDK6, CDK4, RB1, and Cyclin D1 were upregulated in H69AR and H446DDP cells compared to H69 and H446 cells (Figure S1A,B, Supporting Information). To verify the synergistic effect between CDK4/6 inhibitors and chemotherapy drugs, we conducted a synergistic analysis between these drugs. The results showed that both PD and LEE had a synergistic effect with VP16 (Figure 1B). The synergistic effect of PD and LEE with VP16 in H69, H446, and H446DDP are shown in Figure S1C (Supporting Information). To further confirm RNA sequencing results, we performed a western blotting assay and found that CDK6 protein expression was upregulated in H69AR and H446DDP cells compared to H69 and H446 cells but not CDK4 expression (Figure 1C). To uncover the effect of CDK6 on SCLC chemoresistance, we knocked down CDK6 with a CRISPR knockout plasmid (CDK6 sgRNA) and CDK6 siRNA. After transfection with a CDK6 sgRNA plasmid and CDK6 siRNA, we treated the cells with chemotherapeutic drugs, including doxorubicin, cisplatin, and etoposide. The results showed that CDK6 knockout or knockdown in H69AR cells significantly reduced the IC50 value of the chemotherapeutic drugs. Meanwhile, we overexpressed CDK6 using adenovirus in H69 cells, and CDK6 overexpression increased the IC50 value of the chemotherapeutic drugs in H69 cells (Figure 1D). Consistently, CDK6 knockdown decreased the IC50 value in chemotherapy‐treated H446DDP cells, and CDK6 overexpression increased the IC50 value in chemotherapy‐treated H446 cells (Figure 1D). The CDK4/6 inhibitors Palbociclib (PD) and Ribociclib (LEE) are FDA‐approved drugs that are widely used in breast cancer and other cancer types. Subsequently, cell viability was examined in chemotherapeutic‐treated cells, the results indicated that SCLC chemotherapy‐resistant cells are resistant to chemotherapeutic drugs, leading to a higher IC50 value (Figure S1D, Supporting Information). The major effect of CDK4/6 inhibitors is mediating the function of RB1 via mono‐ and subsequent hyperphosphorylation of Cyclin‐CDK complexes. As an RB1 inactivated cell type, SCLC cells seem not to be the target of CDK4/6 inhibitors. To our surprise, PD (0.5 µm) or LEE (0.8 µm) downregulated the IC50 value of chemotherapeutic drugs not only in H69AR and H446DDP cells but also in H69 and H446 cells (Figure 1E). PD and LEE are part of a series of 1‐H‐pyrazole‐3‐carbocamide derivatives and have been evaluated for their CDK inhibitory activities.^[^ ^18^ ^]^ We performed western blot analysis and found that the downregulation of CDK6 protein expression induced by PD or LEE treatment was time‐dependent (Figure 1F). These results suggest that CDK6 participates in the development of chemoresistance in SCLC and that targeting CDK6 may reverse the chemoresistance of SCLC in vitro.

### 2.2 CDK4/6 Inhibitors Inhibit Tumor Growth and Enhance Tumor Response to Chemotherapy in Spontaneous SCLC Mouse Models

**Figure 2:**
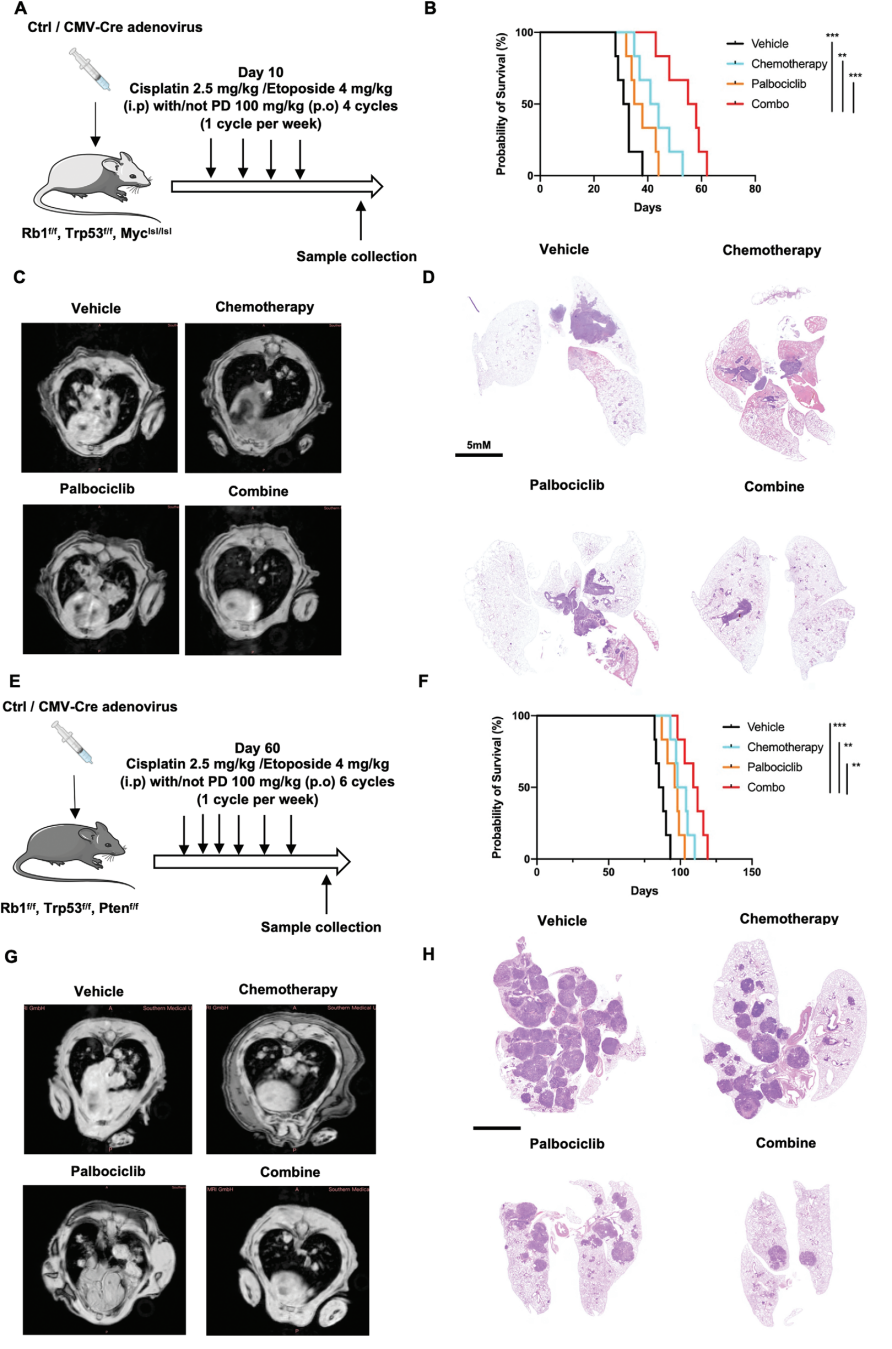
CDK4/6 inhibitors inhibits tumor growth and enhances tumor response to chemotherapy in spontaneous SCLC mouse models. A) Working protocol of chemotherapy (cisplatin 2.5 mg kg^−1^, i.p. injection; etoposide 4 mg kg^−1^, i.p. injection) with or without PD combination (100 mg kg^−1^, administered by gavager on days 1–5, weekly, 1 cycle per week for 4 weeks) in RB1^flox/flox^, Trp53^flox/flox^,Myc^lsl/lsl^ (RPM) mice. B) Kaplan‒Meier survival curve of RPM mice with or without chemotherapy and PD treatment single or combination (*n* = 6). C) MRI images of RPM mice with or without chemotherapy and PD treatment single or combination (*n* = 3). D) HE staining images of RPM mice with or without chemotherapy and PD treatment single or combination. E) Working protocol of chemotherapy (cisplatin 2.5 mg kg^−1^, i.p. injection; etoposide 4 mg kg^−1^, i.p. injection) with or without PD combination (100 mg kg^−1^, administered by gavager on days 1–5, weekly, 1 cycle per week for 6 weeks) in RB1^flox/flox^, Trp53^flox/flox^, Pten^flox/flox^ (RTP) mice (*n* = 6). F) Kaplan‒Meier survival curve of RTP mice with or without chemotherapy and PD treatment single or combination (*n* = 6). G) MRI images of RTP mice with or without chemotherapy and PD treatment single or combination (*n* = 3). H) HE staining images of RTP mice with or without chemotherapy and PD treatment single or combination (*n* = 6). The data are shown as the mean ± SD, **p < 0.01; ***p < 0.001.

To shed light on the effect of CDK4/6 inhibitors in the chemoresistance and chemosenstivity of spontaneous SCLC mouse models, we constructed two different spontaneous SCLC mice models. RB1^flox/flox^, Trp53^flox/flox^, and Myc^lsl/lsl^ (RPM) mice were constructed according to the previously reports. To generate SCLC tumor in those mice, we gave the RPM mice ctrl or CMV‐cre adenovirus administration via tracheal cannula as previously report.^[^ ^19^ ^]^ Ten days after ctrl or CMV‐cre adenovirus administrated, cisplatin (2.5 mg kg^−1^, i.p. injection) and etoposide (4 mg kg^−1^, i.p. injection) treatment with or without PD combination (100 mg kg^−1^, administered by gavager on days 1–5, weekly, 1 cycle per week for 4 weeks) were operated in mice (**Figure** **2**A). Chemotherapy and PD combation group showed better probability of survival compared with chemotherapy group and PD group (Figure 2B). Consistently with the probability of survival, the MRI images showed that the area of tumors in combination groups mice lung was reduced when compared to chemotherapy and PD single treatment groups (Figure 2C). Therefore, we performed HE staining and evaluate the tumor burden in mice lung. Similar to the previously results, the tumors of PTM mice was located near the bronchi^[^ ^19^ ^]^ (Figure 2D). In order to better detect the size of tumor, we used live imaging of small animals to record the mouse lung tumor and carried out area statistic (Figure S2A, Supporting Information). The statistical map of the tumor area in RPM mice is shown in Figure S2B (Supporting Information). Another spontaneous SCLC mice model was constructed in RB1^flox/flox^, Trp53^flox/flox^, and Pten^flox/flox^ (RTP) mice. Differented from the RPM mice, cisplatin (2.5 mg kg^−1^, i.p. injection) and etoposide (4 mg kg^−1^, i.p. injection) treatment with or without PD combination (100 mg kg^−1^, administered by gavager on days 1–5, weekly, 1 cycle per week for 6 weeks) was operated in 60 days after ctrl and CMV‐cre adenovirus administrated (Figure 2E). Chemotherapy and PD combaination group showed better probability of survival than chemotherapy and PD single treatment groups (Figure 2F). It is worth to note that the SCLC tumors in RTP mice were detected in multiple locations in the mice lungs. After MRI images and HE staining analysis, we found that chemotherapy and PD combaination sinifcantly reduced the tumor bunder and tumor size in the mice lung (Figure 2G,H). The statistical map of RTP mice is shown in Figure S2C (Supporting Information). To confirmed the tumors from RPM and RTP mice are neuroendocrine tumors, we stainied CD56 and SYN in these tumors and figures are provided in Figure S2D (Supporting Information).

### 2.3 CDK4/6 Inhibitors Improve Chemosensitivity and Reverse Chemoresistance in Xengrafts SCLC Mice Model

**Figure 3:**
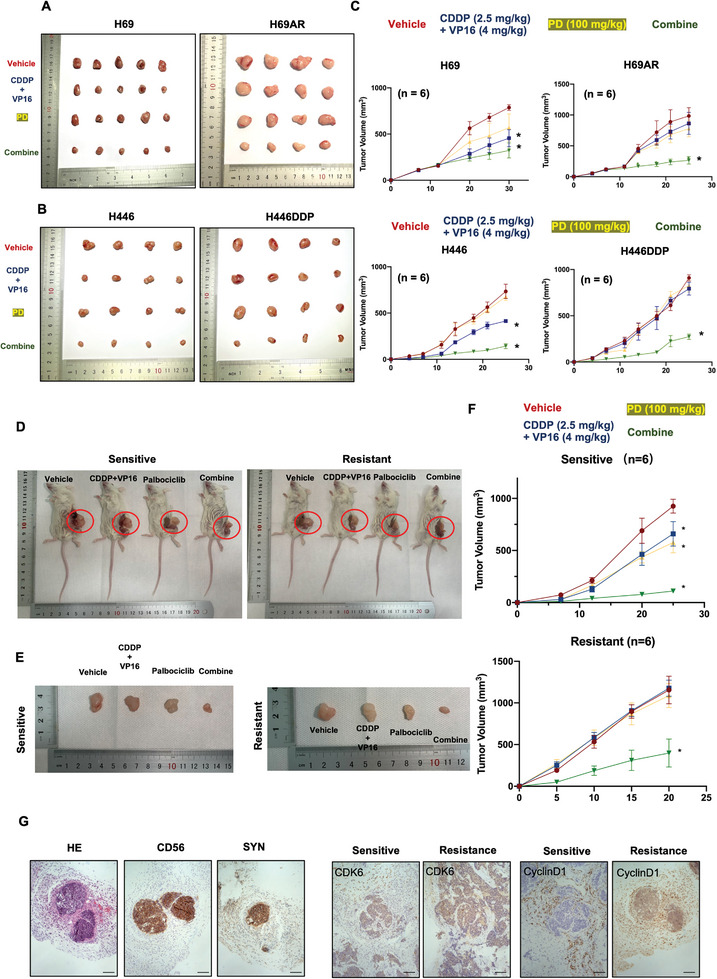
CDK4/6 inhibitors improve chemosensitivity and reverse chemoresistance in xengrafts SCLC mice model. A,B) Images of subcutaneous tumors derived from H69, H69AR, H446, or H446DDP cells after (cisplatin 2.5 mg kg^−1^, i.p. injection; etoposide 4 mg kg^−1^, i.p. injection) with or without PD combination (100 mg kg^−1^, administered by gavager on days 1–5, weekly) are shown (*n* = 6). C) The growth curves of xenografted tumors derived from SCLC cells treated with (cisplatin 2.5 mg kg^−1^, i.p. injection; etoposide 4 mg kg^−1^, i.p. injection) with or without PD combination (100 mg kg^−1^, administered by gavager on days 1–5, weekly) are shown (*n* = 6). D,E) Representative image of chemosensitive and chemotherapy‐resistant PDX model mice that received (cisplatin 2.5 mg kg^−1^, i.p. injection; etoposide 4 mg kg^−1^, i.p. injection), PD treatment (100 mg kg^−1^, administered by gavager on days 1–5, weekly), or combination treatment. The treatment lasted 25 and 20 days respectively. (*n* = 6). F) Tumor growth curves of chemosensitive and chemotherapy‐resistant patient‐derived xenografts after treatment for 25 and 20 days respectively (*n* = 6). G) Left panel. HE, CD56, and SYN staining in SCLC PDX tumors. CD56 and SYN are neuroendocrine markers. Right panel. CDK6 and Cyclin D1 immunostaining in chemosensitive and chemotherapy‐resistant PDX tumors. Scale bar: 50 µm. The data are shown as the mean ± SD, *p < 0.05.

To further confirm the function of CDK4/6 inhibitors in the chemoresistance and chemosensitivity of SCLC in vivo, we performed a tumorigenesis assay by injecting H69 and H69AR cells into the flanks of nude mice and treated the mice with chemotherapeutic drugs (cisplatin, 2.5 mg kg^−1^, i.p. injection; etoposide, 4 mg kg^−1^, i.p. injection) with or without PD (100 mg kg^−1^, administered by gavager on days 1–5, weekly). The results indicated that PD treatment inhibited the tumorigenesis induced by H69 cell injection, and chemotherapy combined with PD significantly inhibited the growth of tumors formed by H69 cells in nude mice (**Figure** **3**A left panel). In chemotherapy‐resistant tumorigenesis induced by H69AR cells injection, chemotherapy or PD single treatment had no effect on the tumor volume in mice. To our surprise, the combination of chemotherapeutic drugs and palbociclib notably reduced the tumor volume induced by H69AR cells in nude mice (Figure 3A right panel). Consistently, we administered chemotherapy combined with PD to nude mice with tumors derived from H446 cells and H446DDP cells, and the results showed that chemotherapy combined with PD obviously reduced tumor volume in these mice (Figure 3B). The statistical analysis of SCLC cell line‐derived xenografts is shown in Figure 3C. We next utilized SCLC PDXs to further investigate the effect of PD on SCLC. To establish a chemotherapy‐resistant PDX model, we treated tumor‐bearing mice with repeated chemotherapy cycles, which mimicked the treatment cycles used in clinical practice in three independent SCLC PDX models as reported previously.^[^ ^7^ ^]^ We treated sensitive and resistant PDX mice with chemotherapy (cisplatin, 2.5 mg kg^−1^, i.p. injection; etoposide, 4 mg kg^−1^, i.p. injection) with or without PD (100 mg kg^−1^, administered by gavager on days 1–5, weekly). The results showed that the combination of PD and chemotherapeutic drugs significantly reduced tumor volume in sensitive and resistant PDX mice (Figure 3D,E). The tumor growth curve are provided in the right panel (Figure 3F). We confirmed the source of the human SCLC tissue by immunostaining for the neuroendocrine markers CD56 and Syn (Figure 3G). Histology analysis indicated that the protein expression and nuclear translocation of CDK6 and Cyclin D1 were increased in resistant PDX mice compared with those in sensitive PDX mice (Figure 3G).

### 2.4 Cell Cycle Modulation Is Not Responsible for the Ability of CDK4/6 Inhibitors to Improve SCLC Chemosensitivity and Autophagic Flux Is Involved in CDK4/6 Inhibitor‐Induced Cell Apoptosis

To determine the effect of CDK4/6 inhibitors in SCLC chemotherapy‐sensitive and chemotherapy‐resistant cells, we performed cell apoptosis analyses in H69 and H69AR cells treated with PD (0.5 µm) or LEE (0.8 µm). Cell death was detected via flow cytometry using an Annexin V‐FITC/PI apoptosis detection kit. The results revealed that PD and LEE treatment promoted cell death in H69 and H69AR cells (Figure S3A, Supporting Information) and the statistical analysis were provided in thr right panel. To further confirm the effect of PD and LEE on cell apoptosis, western blot analysis of the apoptosis markers cleaved PARP and cleaved caspase3 was performed (Figure S3B, Supporting Information) As shown in Figure S3B (Supporting Information), PD (0.5 µm) or LEE (0.8 µm) treatment induced substantial upregulation of cleaved PARP in H69 cells and significant upregulation of cleaved PARP in H69AR cells after 24 h treatment. PD (0.5 µm) or LEE (0.8 µm) treatment reduced the high levels of CDK6 and Cyclin D1 protein expression in H69AR cells. These results suggest that PD (0.5 µm) or LEE (0.8 µm) treatment induces apoptosis in H69 and H69AR cells, which may be one of the triggers by which CDK4/6 inhibitors overcome SCLC chemoresistance. As a master cell cycle regulator, the Cyclin D‐CDK4/6 complex is likely involved in PD (0.5 µm) or LEE (0.8 µm) induced cell apoptosis. Unexpectedly, PD (0.5 µm) or LEE (0.8 µm) treatment had no effect on cell cycle regulation in H69 and H69AR cells (Figure S3D, Supporting Information). The statistical analysis of G1 phase, S phase, and G2 phase provided in the right panel showed no different in cell cycle in H69 and H69AR cells treated with PD (0.5 µm) or LEE (0.8 µm), respectively. As an RB1‐inactive cancer, the protein expression of RB1 in SCLC cells needed to be confirmed. We analyzed RB1 protein expression in our SCLC cell lines H69/H69AR and H446/H446DDP. The results suggested that RB1 protein expression was low in SCLC cell lines and that the cell cycle factors p21 and p27 were not regulated by PD treatment in H69 and H69AR cells (Figure S3E–G, Supporting Information). In Figure S3H (Supporting Information), we used the lung adenocarcinoma cell line A549 as a positive control and confirmed the low expression of RB1 in SCLC cell lines. E2F1 is a critical transcription factor for cell cycle regulation, and we found that E2F1 protein expression was significantly downregulated in H69AR cells compared with H69 cells for unknown reasons (Figure S3I, Supporting Information, up panel). We used the CRISPR Cas9 gene editing strategy to knock down CDK6 in H69AR cells or adenovirus to overexpress CDK6 in H69 cells, and E2F1 protein expression was not changed after CDK6 knockdown or overexpression (Figure 3I low panel). We analyzed E2F1 transcription factor viability using a luciferase assay, and the results suggested that PD (0.5 µm) or LEE (0.8 µm) treatments had no effect on E2F1 promoter viability in H69AR cells, while PD (0.5 µm) or LEE (0.8 µm) significantly inhibited E2F1 promoter viability in A549 cells (Figure S3J, Supporting Information). These results suggest that cell cycle regulation is not involved in the cell apoptosis induced by PD and LEE treatment. We believe that the inactivity of RB1 is one of the reasons.

**Figure 4:**
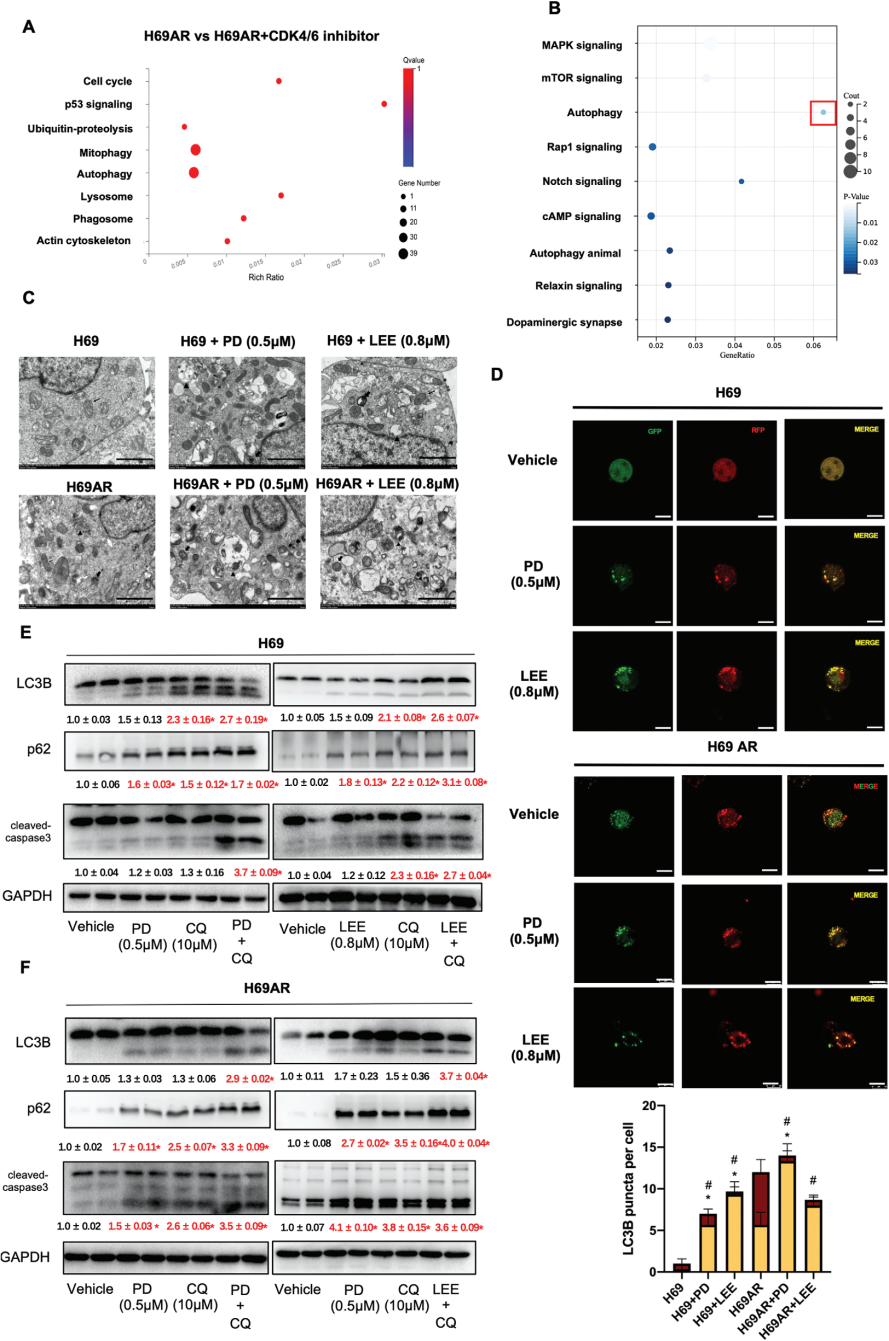
Autophagic flux is involved in CDK4/6 inhibitor‐induced cell apoptosis. A) After RNA sequencing in H69AR cells and H69AR cells treated with CDK4/6 inhibitors, KEGG analysis of differentially expressed genes in bubble plots was performed (the pathway which *p* < 0.05 were shown). B) The Human GeCKOv2A CRISPR knockout pooled library was used to identify genes related to CDK4/6 inhibitors in H69AR cells. KEGG analysis of the negatively regulated genes in H69AR cells treated with CDK4/6 inhibitors was performed (the pathway which *p* < 0.05 were shown). C) Transmission electron microscopy images of H69 and H69AR cells treated with or without PD (0.5 µm) and LEE (0.8 µm). Thin arrow, mitochondria. Thick arrow, autophagosome. Triangle, lysosome. Scale bar: 2 µm. D) Autophagic flux was analyzed in H69 and H69AR cells treated with or without PD (0.5 µm) and LEE (0.8 µm) and transfected with the mRFP‐GFP‐LC3 dual reporter virus (*n* = 6). Scale bar: 10 µm. E,F) Protein expression levels of LC3B, p62 and cleaved caspase‐3 in H69 and H69 AR cells treated with PD and CQ alone or in combination (*n* = 6). The data are shown as the mean ± SD, *p < 0.05. In panel D, *p < 0.05 and ^#^ *p* < 0.05 indicating the different total autophagosomes and impaired autophagosomes between groups, respectively.

The underlying mechanism of PD and LEE induced cell apoptosis in SCLC needed to be explored; thus, we performed RNA sequencing in H69AR and H69AR cells treated with CDK4/6 inhibitors, analyzed the differentially expressed genes, and performed KEGG analysis to determine the possible underlying mechanism of PD and LEE induced SCLC cell apoptosis. Different genes were enriched in autophagy, mitophagy, and lysosome signaling pathways (**Figure** **4**A; Figure S4A, Supporting Information). Mitophagy‐related genes, such as PINK1, PARKIN, and DRP1, were examined via qPCR in PD and LEE treated H69AR cells. The results showed that the mRNA levels of PINK1, PARKIN, and DRP1 were not changed in H69AR cells treated with PD and LEE (Figure S4B, Supporting Information). The Human GeCKOv2A CRISPR knockout pooled library was used to identify genes related to CDK4/6 inhibitors in H69AR cells. After the Cas9‐sgRNA library was constructed in H69AR cells, we treated mutant H69AR cells with vehicle or PD (0.5 µm) for 7 days to enable positive and negative screening. Then, we found that negative genes were enriched in the autophagy signaling pathway (Figure 4B). These results suggested that autophagy may be involved in CDK4/6 inhibitor‐induced cell apoptosis. To further confirm these results, we analyzed autophagy in H69 and H69AR cells treated with PD (0.5 µm) or LEE (0.8 µm) via transmission electron microscopy (TEM). Representative images showed that autophagosomes were increased, mitochondria were destroyed, and lysosomes were dysfunctional in H69 and H69AR cells treated with PD and LEE and that the number of autophagosomes was larger than that of autophagolysosomes (Figure 4C). These results suggestted that PD (0.5 µm) or LEE (0.8 µm) treatment induced autophagy and that autophagic flux is impaired (Figure 4C; Figure S4C, Supporting Information). As many other studies have reported, impaired autophagic flux is critically involved in many cell events. To detect autophagic flux, we transfected H69, H69AR, H446, and H446DDP cells with an mRFP‐GFP‐LC3 dual reporter virus with or without PD (0.5 µm) or LEE (0.8 µm) treatment. The results showed that the autophagy level was improved in H69AR cells compared with H69 cells and that the high level of autophagy may be a positive regulator of cell viability and may be involved in SCLC cell chemoresistance, as we previously reported.^[^ ^20^ ^]^ PD (0.5 µm) or LEE (0.8 µm) treatment induced double‐positive LC3B puncta in SCLC cells, which indicated that PD and LEE treatment induced impaired autophagic flux in SCLC cells (Figure 4D; Figure S4D, Supporting Information). To confirm that impaired autophagic flux is one of the triggers of PD and LEE induced cell apoptosis, we treated H69 and H69AR cells with PD(0.5 µm) or LEE (0.8 µm) and CQ (10 µm), a terminal‐phase inhibitor of autophagosome‐lysosome fusion. Western blot results suggested that both CQ and CDK4/6 inhibitors promoted LC3B and p62 protein expression in H69 and H69AR cells. The combination of CQ and CDK4/6 inhibitors significantly upregulated the protein expression levels of cleaved PARP and cleaved caspase‐3. These results suggest that impaired autophagic flux induced by CQ or CDK4/6 inhibitors promotes SCLC cell apoptosis (Figure 4E,F). We found that CDK4/6 inhibitors combined with CQ increased the protein expression of autophagy markers LC3B and p62 in H69 and H69AR cells, we also detected cleaved‐caspase3 as a cell death marker in these experiments and found that cleaved‐caspase3 protein expression upregulated in CDK4/6 inhibitors + CQ group (Figure 4F). Considering the above results, we further wondered whether gene editing of CDK6 could affect autophagy. We used CRISPR Cas9 and siRNA to knock down CDK6 in H69AR cells and used adenovirus to overexpress CDK6 in H69 cells and analyzed autophagy‐related proteins, such as Beclin1, ATG5, p62, and LC3B (Figure S4E, Supporting Information). Western blotting results showed that CDK6 downregulation induced impaired autophagic flux in H69AR cells and that CDK6 overexpression in H69 cells promoted autophagy induction, with a smooth autophagic flux. Real time PCR results showed the mRNA levels of CDK6, CDK4, CyclinD, Ambra1, Beclin, ATG5, p62, and LC3B were examined in H69, H69AR, and H69AR cells treated with PD (0.5 µm) or LEE (0.8 µm) for 24 h (Figure S4F, Supporting Information). In general, CDK6 is involved in autophagic flux regulation, and impaired autophagic flux damages chemotherapy‐resistant SCLC cells.

### 2.5 Lysosomal Dysfunction Mediated by CDK4/6 Inhibitors

**Figure 5:**
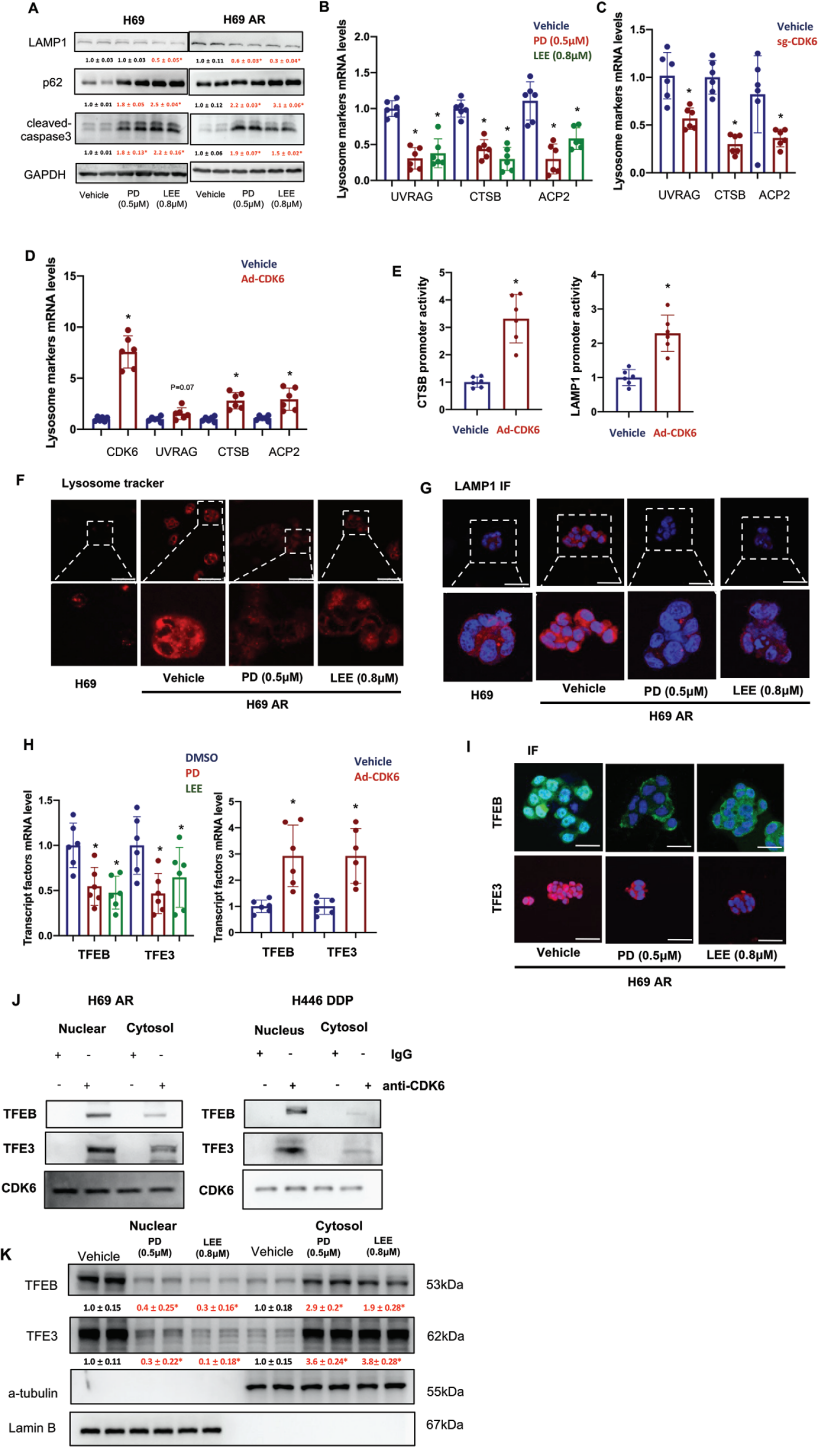
Lysosomal dysfunction mediated by CDK4/6 inhibitors. A) Protein expression levels of LAMP1, p62 and cleaved caspase‐3 were detected in H69 and H69AR cells with or without PD (0.5 µm) and LEE (0.8 µm) treatment (*n* = 6). B) Lysosome‐associated genes (UVRAG, CTSB, and ACP2) were detected in H69AR cells treated with PD (0.5 µm) and LEE (0.8 µm) (*n* = 6). C,D) UVRAG, CTSB, and ACP2 mRNA levels in CDK6 sgRNA‐transfected H69AR and CDK6‐overexpressing adenovirus‐infected H69 cells (*n* = 6). E) CTSB and LAMP1 promoter activities in blank vector‐infected and CDK6‐overexpressing adenovirus‐infected H69 cells (*n* = 6). F,G) Images of lysosome marker and LAMP1 immunofluorescence in H69 and H69AR cells with or without PD and LEE treatment (*n* = 3). Scale bar: 10 µm. H) mRNA levels of TFEB and TFE3 in H69AR cells treated with PD and LEE or in H69 cells infected with CDK6‐overexpressing adenovirus (*n* = 6). I) Immunofluorescence images of TFEB and TFE3 in H69AR cells with or without PD (0.5 µm) and LEE (0.8 µm) treatment. Scale bar: 10 µm. J) Immunoprecipitation of CDK6 with TFEB and TFE3 in the nucleus and cytoplasm of H69AR and H446DDP cells. K) Protein expression levels of TFEB and TFE3 in the nucleus and cytoplasm of H69AR cells with or without PD and LEE treatment (*n* = 6). The data are shown as the mean ± SD, *p < 0.05.

As many other studies have suggested, lysosomal dysfunction is one of the critical triggers involved in autophagic flux stagnation. To determine whether lysosomal dysfunction is involved in the autophagy stagnation mediated by CDK4/6 inhibitors, we conducted western blot analysis and found that PD and LEE treatments downregulated the lysosome‐related protein LAMP1 and increased p62 protein expression, an autophagy stagnation marker. We used cleaved‐caspase3 to confirm the cell damage effect mediated by PD (0.5 µm) or LEE (0.8 µm) (**Figure** **5**A). Certain lysosome‐related markers (UVRAG, CTSB, and ACP2) were detected in H69AR cells treated with PD and LEE or transfected with sg‐CDK6. In Figure 5B,C, pharmacological inhibition of CDK6 or CDK6 knockout mediated by gene editing significantly reduced the mRNA levels of UVRAG, CTSB, and ACP2. On the other hand, CDK6 overexpression induced upregulation of UVRAG, CTSB, and ACP2 mRNA levels (Figure 5D). CTSB promoter viability and LAMP1 promoter viability were detected using luciferase assays in CDK6‐overexpressing H69 cells. The results showed that CDK6 overexpression increased CTSB and LAMP1 promoter viability in H69 cells (Figure 5E). To confirm the effect of PD and LEE on lysosomal function, we performed lysosome tracker staining and LAMP1 immunofluorescence in H69, H69AR, H446, and H446DDP cells treated with or without PD (0.5 µm) or LEE (0.8 µm). The results indicated that the fluorescence intensity of lysosome tracker and LAMP1 was higher in H69AR cells than in H69 cells, while PD and LEE treatments notably decreased the fluorescence intensity of lysosome tracker and LAMP1 (Figure 5F,G; Figure S5A, Supporting Information). Furthermore, we found that LAMP1 immunofluorescence intensity was higher in CDK6‐overexpressing H69 cells than in vehicle‐treated H69 cells and that CDK6 knockout/knockdown H69AR cells exhibited lower LAMP1 intensity than vehicle‐treated H69AR cells (Figure S5B, Supporting Information). Previous studies have suggested that TFEB and TFE3 are master lysosome biogenesis regulators in a number of biological processes. To further confirm whether TFEB and TFE3 are involved in the mechanism by which CDK4/6 inhibitors mediate lysosomal function deletion, TFEB and TFE3 mRNA levels were detected. PD and LEE treatments decreased (in H69AR cells) and CDK6 overexpression increased the mRNA level of TFEB or TFE3 in H69 cells (Figure 5H). Nuclear translocation is critical during TFEB‐ and TFE3‐mediated lysosome function. We performed an immunofluorescence assay in H69AR and H446DDP cells and found that PD and LEE treatments decreased the nuclear translocation of TFEB and TFE3 (Figure 5I; Figure S5C, Supporting Information). Then, we performed TFEB and TFE3 immunofluorescence assays in CDK6‐overexpressing H69 cells and CDK6 knockout/knockdown H69AR cells. Images showed that CDK6 overexpression in H69 cells promoted TFEB and TFE3 nuclear translocation and that CDK6 knockdown or knockout significantly inhibited TFEB and TFE3 nuclear translocation (Figure S5D, Supporting Information). To explore the relationship between CDK6 inhibition and TFEB and TFE3 nuclear translocation, we performed a coimmunoprecipitation assays, and the results showed that CDK6 interacted with TEFB and TFE3 in the cytosol and nucleus (Figure 5J; Figure S5E, Supporting Information). Western blot analysis (Figure 5K) showed that PD (0.5 µm) or LEE (0.8 µm) treatments increased the expression of TFEB and TFE3 in the cytosol and decreased the expression of TFEB and TFE3 in the nucleus. Although the mechanism by which CDK6 regulates TFEB and TFE3 is not clear, our results provide evidence that CDK6 regulates lysosomal function by mediating the cytosolic translocation of TFEB and TFE3. These may be the underlying mechanisms by which CDK4/6 inhibitors mediate lysosomal function deletion and subsequent autophagy stagnation.

### 2.6 AMBRA1 as a Target of CDK4/6 Inhibitors in SCLC Cells

**Figure 6:**
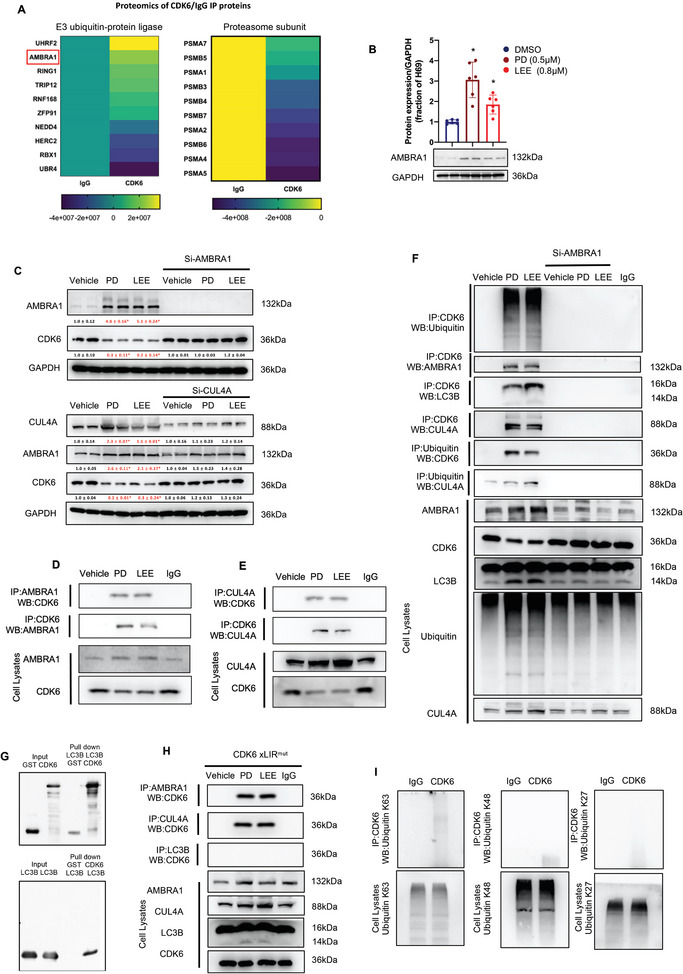
AMBRA1 is a target of CDK4/6 inhibitors in SCLC cells. A) Proteomic analysis of CDK6‐interacting proteins in H69AR cells treated with PD.B) AMBRA1 protein expression in H69AR cells with or without PD (0.5 µm) and LEE (0.8 µm) treatment. C) Protein expression of AMBRA1 and CDK6 in H69AR cells or siAMBRA1‐transfected H69AR cells with or without PD (0.5 µm) and LEE (0.8 µm) treatment. CUL4A, AMBRA1, and CDK6 protein expression in H69AR cells or siCUL4A‐transfected H69AR cells with or without PD and LEE treatment (*n* = 6). D,E) Immunoprecipitation of CDK6 with AMBRA1 and CUL4A in PD‐ and LEE‐treated H69AR cells. F) Immunoprecipitation of CDK6 with ubiquitin, AMBRA1, LC3B, and CUL4A in scramble‐ or siAMBRA1‐transfected H69AR cells with or without PD and LEE treatment. G) Direct interaction between CDK6 and LC3B confirmed by GST pull‐down assays. H) xLIR domain mutation inhibited the CDK6 and LC3B interaction in PD (0.5 µm) and LEE (0.8 µm) treated H69AR cells. I) Immunoprecipitation of CDK6 with K27 ubiquitin, K48 ubiquitin and K63 ubiquitin in PD‐treated H69AR cells. CDK6 is ubiquitinated by K63 ubiquitin and K48 ubiquitin but not K27 ubiquitin. The data are shown as the mean ± SD, *p < 0.05.

In addition to the above autophagy studies, we focused on the mechanism by which CDK4/6 inhibitors mediate CDK6 protein degradation. We performed a proteomics assay on the proteins interacting with IgG and CDK6 in H69AR cells (**Figure** **6**A). In these results, we found that CDK6 interacted with 10 E3 ubiquitin‐protein ligases, including UHRF2, AMBRA1, RING1, and TRIP12, among other molecules. Furthermore, CDK6 could not interact with certain proteasome subunits, such as PSMA7, PSMB5, and other proteasome subunits. Previous studies have suggested that AMBRA1 is the main regulator of Cyclin complex stability. AMBRA1 deficiency results in high levels of Cyclin D in cells and promotes cell proliferation. Mechanistically, AMBRA1 acts as a regulator of the Cullin4 E3 ligase complex and mediates ubiquitylation and proteasomal degradation of the Cyclin D complex. Western blot analysis suggested that PD (0.5 µm) treatment induced approximately threefold AMBRA1 protein expression compared to the DMSO group and that LEE (0.8 µm) induced nearly twofold AMBRA1 protein expression compared to DMSO (Figure 6B). Similar experiments were performed in H446DDP cells, and PD and LEE treatments increased AMBRA1 protein expression in H446DDP cells (Figure S6A, Supporting Information). To further validate the effect of AMBRA1 on CDK6 protein degradation induced by CDK4/6 inhibitors, we knocked down AMBRA1 with siRNA and treated the cells with PD and LEE. The western blot analysis in Figure 6C shows that PD (0.5 µm) and LEE (0.8 µm) induced AMBRA1 upregulation and CDK6 downregulation. When AMBRA1 was knocked down with siRNA, PD and LEE had no effects on CDK6 protein expression (Figure 6C). AMBRA1 is a critical regulator of the Cullin4 E3 ligase complex, and CUL4A is thought to be another critical regulator involved in AMBRA1‐mediated CDK6 protein expression. After CUL4A knockdown, we found that PD (0.5 µm) and LEE (0.8 µm) barely increased AMBRA1 protein expression and had no effects on CDK6 protein expression, similar to AMBRA1 knockdown (Figure 6C). To reveal the relationship between AMBRA1 and CDK6, we performed an immunofluorescence assay and confirmed that CDK6 colocalized with AMBRA1 in PD‐treated H69 AR cells (Figure S6B, Supporting Information). Next, we performed a coimmunoprecipitation assays and found that PD and LEE treatments not only induced the interaction between AMBRA1 and CDK6 protein (Figure 6D) but also induced the interaction between CUL4A and CDK6 protein in H69AR and H446DDP cells (Figure 6E; Figure S6C, Supporting Information). As E3 ubiquitin ligases, AMBRA1 and CUL4A enhance protein ubiquitination and degradation. To confirm whether CDK6 is degraded via this pathway, we performed a coimmunoprecipitation assay in AMBRA1 knockdown H69AR cells. Western blot analysis revealed that AMBRA1 knockdown significantly inhibited the interaction of CDK6 with ubiquitin, AMBRA1, CUL4A, and LC3B induced by PD and LEE. The interactions between CDK6 and ubiquitin, CUL4A and LC3B were enhanced by PD (0.5 µm) and LEE (0.8 µm) in H69AR cells but not in AMBRA1 knockdown H69AR cells (Figure 6F). To confirm that CDK6 is involved in AMBRA1‐mediated protein degradation, we performed a GST pull‐down assay in a cell‐free system. The results demonstrated that CDK6 interacts with LC3B directly and that PD and LEE likely downregulate CDK6 protein expression by mediating the AMBRA1‐CUL4A‐LC3B autophagy pathway (Figure 6G). However, the domain through which CDK6 interacts with autophagosomes was still not clear. Thus, we performed iLIR analysis and found an xLIR domain in the CDK6 protein (96‐101 aa, LVFEHV; Figure S6D, Supporting Information). To validate the putative interaction domain of CDK6, we constructed a CDK6 xLIR domain mutation plasmid, and transfected H69AR cells with the plasmid. After single‐clone selection, we treated H69AR‐xLIRmut cells with PD and LEE and performed a coimmunoprecipitation assay. As shown in Figure 6H, we found that PD and LEE treatments induced the interaction of CDK6 with AMBRA1 and CUL4A but not LC3B in H69AR‐xLIRmut cells. PD and LEE treatments did not downregulate CDK6 protein expression in H69AR‐xLIRmut cells. As many other studies have suggested, different ubiquitin linkage types serve as platforms that trigger other signal transduction pathways.^[^ ^21^ ^]^ In our study, we sought to determine which ubiquitin linkage types are involved in AMBRA1‐mediated CDK6 protein degradation and found that CDK6 interacted with K63‐ubiquitin and K48‐ubiquitin but not K27‐ubiquitin (Figure 6I). These results suggest that CDK4/6 inhibitors induce downregulation of CDK6 protein expression via AMBRA1‐CUL4A‐ubiquitin and the autophagy pathway and that the key domain in CDK6 by which CDK6 degradation is regulated is at amino acids 96–101.

### 2.7 CDK6 Expression Is Upregulated in SCLC Tissues and Predicts a Poor Prognosis

**Figure 7:**
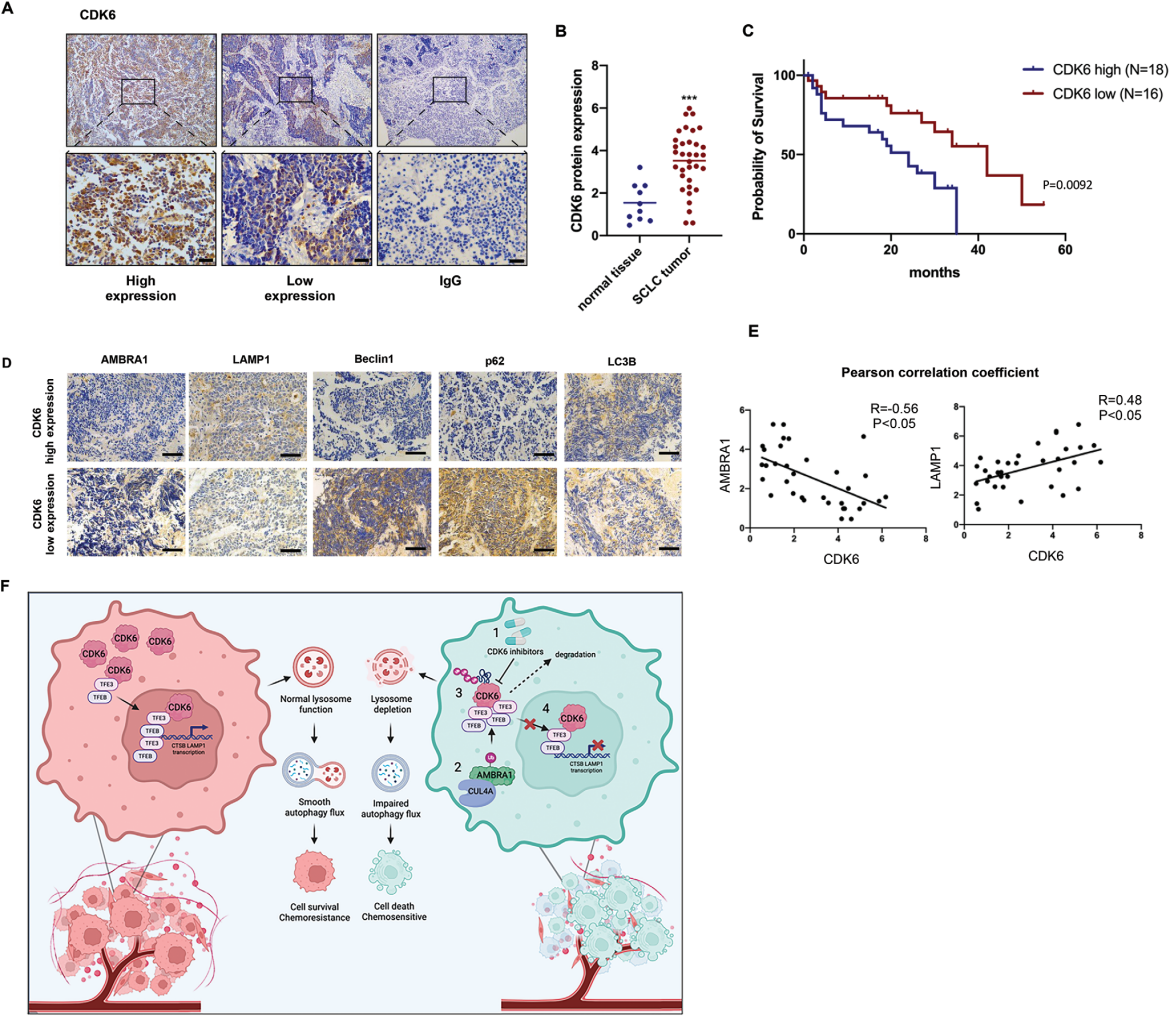
CDK6 expression is upregulated in SCLC tissues and predicts a poor prognosis. A) Immunohistochemistry images showing CDK6 expression in paraffin tissues from SCLC patients (*n* = 34). The left panel is a representative picture of patient tissue with high CDK6 expression. The center panel is a representative picture of patient tissue with low CDK6 expression. Scale bar: 50 µm. B) Differential CDK6 expression was compared between SCLC tissue samples (*n* = 34) and matched normal tissue samples (*n* = 10). The median CDK6 expression level in each group is represented by a horizontal line in the scatter plot. C) Kaplan‒Meier survival curve showing the correlation between CDK6 expression and overall survival in 34 SCLC patients. D) Immunohistochemistry images of AMBRA1, LAMP1, Beclin1, p62, and LC3B expression in tumors with high CDK6 expression and tumors with low CDK6 expression (*n* = 34). Scale bar: 50 µm. E) Pearson correlation coefficient results for CDK6‐AMBRA1 and CDK6‐LAMP1 (*n* = 34). F) A signaling pathway map illustrating how CDK4/6 inhibitors ameliorate SCLC chemotherapy resistance by regulating autophagy. The data are shown as the mean ± SD, ****p < 0.001.

We further investigated the relationship between increased CDK6 protein expression and the prognosis of patients with SCLC. To assess the CDK6 protein expression level in SCLC patients, we performed CDK6 immunohistochemistry (IHC) on biopsy samples, including 10 normal lung tissues and 34 SCLC tumors (**Figure** **7**A). As shown in Figure 7B, CDK6 expression was significantly increased in 34 SCLC patient tissues compared with matched nontumor tissue samples (Figure 7B and **Table** **1**). Furthermore, a Kaplan‒Meier survival curve showed that SCLC patients with higher CDK6 expression had poorer overall survival than SCLC patients with lower CDK6 expression (Figure 7C). We further analyzed AMBRA1, LAMP1, Beclin1, p62, and LC3B protein expression in the CDK6 high expression group and CDK6 low expression group (Figure 7D). Through CDK6, AMBRA1, and LAMP1 IHC staining, we observed a significant negative correlation between CDK6 and AMBRA1 expression and a positive correlation between CDK6 and LAMP1 expression in tissue samples (Figure 7E). As shown in Figure S7 (Supporting Information), patients with higher CDK6 expression had poorer survival than patients with lower CDK6 expression in EGAS00001000925 (Figure S7A, Supporting Information). A negative correlation between CDK6 and AMBRA1 and a positive correlation between CDK6 and LAMP1 were confirmed in the GSE149507 database (Figure S7B, Supporting Information). Based on these results, we conclude that CDK4/6 inhibitors may regulate CDK6 degradation via the AMBRA1‐CUL4A complex and negatively impact lysosomal function via TFEB and TFE3, followed by impaired autophagy and SCLC cell death (Figure 7F).

**Table 1:** Clinical characteristics of 34 patients with SCLC according to the protein expression of CDK6.

Variable	CDK6		*p* value
	High	Low	
Age, Year, ≤62: >62	10:8	10:6	0.7385
Sex, male: female	14:4	12:4	>0.999
Smoking, yes: no	11:7	10:6	>0.999
Disease stage, LD:ED	3:15	11:5	0.0045**
Status, survival: death	0:18	5:11	0.0157*

## 3 Discussion

In this study, we found a novel effect of CDK4/6 inhibitors on SCLC chemoresistance. We demonstrated that CDK4/6 inhibitors impeded chemoresistance and inhibits tumor growth using different SCLC animal models. After RNA sequencing and proteomics were performed, we found that CDK4/6 inhibitors mediated autophagic flux in SCLC by regulating AMBRA1 activity. Previous studies have shown that AMBRA1 regulates the stability of Cyclin D via the E3 ubiquitin signaling pathway.^[^ ^17^, ^22^ ^]^ Consistently, we found that AMBRA1 was a downstream target of CDK4/6 inhibitors and induced CDK6 protein degradation via ubiquitination. Although the underlying mechanism of CDK6 protein degradation was not clearly provided, our results suggest that CDK6 protein interacts with E3 ubiquitin ligase but not proteasome subunits (Figure 6A). The high Cyclin D levels may be the reason of CDK6 protein stabilization. In part of CRL4 ubiqutin ligase complex, phosphorylated AMBRA1 can interact with NUMA1 and induced NUMA1 proper localization at the cell cortex. And these phosphorylation events mediated by kinases such as CDK1 may be the trigger of AMBRA1 activation. Some other studies also suggest AMBRA1 act as the regulator of other CDKs stabilization.^[^ ^23^ ^]^ These results suggest that CDK6 protein degradation likely occurs via the autophagy signaling pathway. Furthermore, through proteomics, Co‐IP and GST pull‐down assays of CDK6 and LC3B, we found that CDK6 protein was degraded via autophagy signaling. The direct interaction between CDK6 and LC3B allows CDK6 protein to be degraded via autophagosomes. Notably, we identified an LC3B binding site (96‐101 AA) in the CDK6 protein by using an xLIR system.^[^ ^24^ ^]^ After mutation of the xLIR motif in CDK6, the interaction between CDK6 and AMBRA1 or CUL4A was not affected. However, the interaction between CDK6 and LC3B could not be detected in CDK6 xLIR mutant H69AR cells. These results indicate that autophagy is involved in cell cycle regulation in chemotherapy‐resistant SCLC cells. Notably, SCLC is an RB1‐inactivated cancer that is not listed as a candidate for CDK4/6 inhibitors. To further understand the biological function of CDK4/6 inhibitors in the chemoresistance of SCLC, we initially discovered that CDK4/6 inhibitors cannot regulate the cell cycle in RB1‐inactivated chemotherapy‐resistant and chemosensitive SCLC cells.

After the Cyclin‐dependent kinase (CDK) holoenzyme was identified as a key trigger of cell cycle transition, CDK inhibitors became a promising therapeutic option. However, pan‐CDK inhibitors are not widely used due to their dose‐limiting toxicities.^[^ ^25^ ^]^ Currently, the new generation of CDK4/6 inhibitors (palbociclib, abemaciclib, and ribociclib) have become broadly used cancer therapeutics.^[^ ^26^ ^]^ After the striking success of combining CDK4/6 inhibitors and the hormone receptor antagonist letrozole for breast cancer treatment, many other CDK4/6 inhibitors are being evaluated in ongoing clinical trials.^[^ ^27^ ^]^ Thus, the underlying mechanism of the antitumorigenic effects of CDK4/6 inhibitors needs to be determined. As a bridge between numerous extracellular signaling pathways and the cell cycle,^[^ ^28^ ^]^ CDK4/6 mainly depends on the phosphorylation and inactivation of the retinoblastoma tumor‐suppressor protein RB1. Although RB1 is the primary cell cycle target of CDK4/6, other studies have suggested that non‐RB1 targets might be involved in the immune‐related, senescence promoting and metabolic reprogramming activity of CDK4/6 inhibitors.^[^ ^29^ ^]^

As found in many other studies, autophagic flux disorder is common in SCLC chemoresistance.^[^ ^15^, ^20^ ^]^ Compared with chemosensitive SCLC cells, chemotherapy‐resistant SCLC cells show higher levels of autophagy and better lysosomal function. Other studies have suggested that lysosomal biomass may be responsible for the resistance of other cancer types to CDK4/6 inhibition.^[^ ^30^ ^]^ This may protect chemotherapy‐resistant SCLC cells during chemotherapeutic drug treatment. Similar results have been reported in other studies showing that autophagy inhibition reverses chemoresistance in SCLC^[^ ^31^ ^]^ and that increasing the pH of lysosomes to reduce lysosomal function may be an option for SCLC treatment.^[^ ^32^ ^]^ In the present study, we found that CDK6 expression was associated with TFEB/TFE3 translocation. A number of studies have suggested that TFEB/TFE3 are master regulators in lysosome function modulation.^[^ ^33^ ^]^ Our results suggest that AMBRA1‐induced ubiquitination of CDK6 protein leads to TFEB/TFE3 cytosolic translocation. A previous study showed that chemical or genetic inactivation of CDK4/6 increased lysosomal numbers by activating TFEB/TFE3.^[^ ^34^ ^]^ We found that chemoresistance resulted in TFEB/TFE3 nuclear translocation and that CDK4/6 inhibitors or genetic inactivation of CDK4/6 led to TFEB/TFE3 cytosolic translocation. This finding may provide new insight into chemoresistance development in SCLC cells. We believe that TFEB/TFE3 translocation is cell‐type specific and may play different roles in cell cycle regulation. Overall, lysosomal biomass is critical in chemoresistance formation. Chemical or genetic inactivation of CDK6 abolished lysosomal function, which was followed by autophagy stagnation.

As an important kinase that regulates cell cycle and basic cell behavior, CDK6 is equally important in other biological functions. And our findings may be part of these already observed biological processes. CDK6 expression may mediating angiogenesis in tumors which may contributed to cancer cell survival during chemotherapy.^[^ ^35^ ^]^ Other results suggested that CDK6 protein expression involved in DNA repair which may be possible reason to induced autophagy.^[^ ^36^ ^]^ Some reports suggested the the stability of CyclinD1 may be the limiting factor of autophagy activation.^[^ ^37^ ^]^ During chemoresistance or CDK4/6 inhibitors treatment, epigenetic mechanisms also involved in chemoresistance formation and chemotherapy.^[^ ^38^ ^]^ Some literature on immunomodulation with CDK4/6 inhibitors suggested that Trilaciclib enhance and prolong the duration of the antitumor response by chemotherapy/ICI combinations.^[^ ^13^ ^]^ Taken together, all these results suggested a close relationship between CDK4/6 inhibitors treatment and autophagy activation. These results may provided us new insight in cancer therapy.

In summary, we provided solid evidence that CDK4/6 inhibitors suppress chemoresistance in SCLC. We also demonstrated that deletion of lysosomal function by CDK4/6 inhibitors led to the death of chemotherapy‐resistant SCLC cells. For the first time, we provided evidence that CDK4/6 inhibitors overcome chemoresistance in SCLC cells and verified that CDK4/6 inhibitors play an important role in lysosomal activity. These findings provide a basic mechanism supporting the use of CDK4/6 inhibitors in SCLC patients, which may be a possible therapeutic method to reverse chemoresistance in SCLC.

## 4 Experimental Section

### Human Tissue Specimens from SCLC Patients

Paraffin‐embedded tumor sections from 34 patients with small cell lung cancer were collected from Fujian Provincial Hospital. These patients received fiberoptic bronchoscopy or biopsy from January 2013 to September 2016 and received follow‐up care at Fujian Provincial Hospital. All patients provided informed consent before specimen collection, and this study was conducted in accordance with the Declaration of Helsinki.

### Cell Lines and Cell Culture

The NCI‐H69, NCI‐H69AR, and NCI‐H446 cell lines were purchased from the American Type Culture Collection (ATCC, USA). A drug‐resistant subtype of NCI‐H446 cells, named NCI‐H446DDP cells, were established by culturing H446 cells in complete medium containing gradually increasing concentrations of cisplatin (Hausen Pharmaceutical, China) from 0.2 to 0.6 µg mL^−1^ for 12 months. All cell lines were maintained in RPMI medium (HyClone, USA) supplemented with 15% fetal bovine serum (Gibco, USA) and were incubated at 37 °C in a humidified atmosphere with 5% CO2. The cell lines used in this study were mycoplasma‐free and were routinely verified by morphological quality examinations and the growth profile. Palbociclib (PD) 0.5 µm and Ribociclib (LEE) 0.8 µm were used in the cell culture experiments to examine the function of CDK4/6 inhibitors in chemoresistant formation.

### Cell Transfection

Cells were transiently transfected with validated siRNAs targeting CDK6, CUL4A, or AMBRA1 and with the corresponding negative control siRNA (GenePharma, China). (siCDK6 sequence: 5′‐GCAGAAATGTTTCGTAGAA‐3′, siAMBRA1 sequence: 5′‐GCCAGUAACAUUGCCAAUATT‐3′, siCUL4A sequence: 5′‐CCAUCUGGGAUAUGGGAUUTT‐3′). Cells were infected with adenovirus encoding CDK6 (Ad‐CDK6) and transfected with a 96–101 amine acid mutation CDK6 plasmid (Genechem, China). Cells were harvested at the indicated time points for further analysis. Lipofectamine 3000 Transfection Reagent (Invitrogen, USA) was used for siRNA and plasmid transfection.

### Synergy Effect of CDK4/6 Inhibitors Combined with Chemotherapy Drugs

Different concentration of CDK4/6 inhibitors Palbociclib (PD) and Ribociclib (LEE) treated the chemosensitive and chemoresistant SCLC cell lines H69, H69AR, H446, and H446DDP. Cell viability was detected by cell counting Kit‐8 assay and the synergy effect of CDK4/6 inhibitors combined with chemotherapy drugs are analyzed by Synergyfinder (http://synergyfinder.fimm.fi).

### CRISPR‐Cas9 Knockout of CDK6

Knockout of CDK6 in H69AR cells was performed using CRISPR/Cas9‐guided genome editing technology. Briefly, a 20‐nucleotide sgRNA sequences: 5′‐CCGCCACGCATTCGTACTGC‐3′ (92462583‐92462603) was designed using the sgRNA CRISPR design tool online (http://www.e‐crisp.org) and cloned into a pSpCas9(BB)−2A‐puro (PX459) plasmid (Addgene, USA). After cloning, plasmids were purified and verified by sequencing. H69AR cells were transfected with CDK6 sgRNA plasmid using Lipofectamine 3000 (Invitrogen, USA) according to the manufacturer's instructions. Puromycin was added to select the transfected cells after 48 h of transfection. Knockout efficiency for CDK6 was assessed via western blotting.

### Cell Counting Kit‐8 Assay

The drug resistance of cells was determined using Cell Counting Kit‐8 (Dojindo, Japan) assays. Cells in complete medium were seeded into 96‐well plates at a density of 5000–10 000 cells per well. After 24 h of culture, chemotherapeutic drugs were added to the wells, including cisplatin (Hausen Pharmaceutical, China), etoposide (Vepesid, Australia), and doxorubicin (Pfizer, USA). Wells without drug were used as controls. After 24 h of treatment, CCK8 working solution (Dojindo, Japan) was added to the plates, followed by further incubation for 1–4 h. The OD at 450 nm was measured, and the 50% inhibitory concentration (IC50) was calculated using GraphPad. Each experiment was performed in triplicate.

### Quantitative Real‐Time RT‐PCR

Total RNA was extracted using TRIzol reagent (Takara, Japan) and then reverse transcribed with a Fast Quant RT kit (TIANGEN, China). The resulting complementary DNA was quantified via real‐time PCR using Talent qPCR PreMix (TIANGEN, China) on a Bio‐Rad CFX Connect instrument. The sequences of the primers are listed in Supporting Information.

### Western Blotting and Coimmunoprecipitation (Co‐IP) Assay

Cells were lysed in RIPA lysis buffer (CWBIO, China) supplemented with protease and phosphatase inhibitor cocktail (CWBIO, China) for 30 min at 4 °C. Immunoblotting was performed via electrophoresis and incubation with primary antibodies against LC3B (1:1000, 2775S, Cell Signaling Technology, USA), CDK6 (1:1000, ab124821, Abcam, UK), CDK4 (1:1000, 12790T, Cell Signaling Technology, USA), RB1 (1:200, 554 145, BD Biosciences, USA), p62 (1:1000, AP6006, Bioworld Technology, USA), ATG5 (1:1000, AP6026, Bioworld Technology, USA), Beclin1 (1:1000, T55092S, Abmart Biomedicine, China), CUL4A (1:1000, T58391, Abmart Biomedicine, China), AMBRA1 (1:1000, 13762‐1‐AP, Proteintech Group, USA), LAMP1 (1:1000, 55273‐1‐AP, Proteintech Group, USA), TFEB (1:200, sc‐166736, Santa Cruz Biotechnology, USA), TFE3 (1:1000, 14480‐1‐AP, Proteintech Group, USA), Ubiquitin (1:1000, 10201‐2‐AP, Proteintech Group, USA), Ubiquitin‐K27 (1:1000, ab181537, Abcam, UK), Ubiquitin‐K48 (1:1000, ab140601, Abcam, UK), Ubiquitin‐K63 (1:1000, ab179434, Abcam, UK), E2F1 (1:1000, PAB44204, ZEN‐BIOSCIENCE, China), Cyclin D1 (1:1000, ab16663, Abcam, UK), p21 (1:1000, ab109520, Abcam, UK), p27 (1:1000, ab32034, Abcam, UK), PARP1 (1:1000, 13371‐1‐AP, Proteintech Group, USA), Cleaved PARP (1:1000, 5625S, Cell Signaling Technology, USA), and Caspase‐3 (1:1000, 14220S, Cell Signaling Technology, USA). Following incubation with HRP‐conjugated secondary antibodies, the blots were visualized using chemiluminescence reagent (Millipore, USA) in an ECL detection system. Densitometric analysis was performed using ImageJ software. Each experiment was repeated three times. Coimmunoprecipitation (Thermo Scientific, USA) assays were used in this study. Cell samples subjected to an IP assay were immunoprecipitated with the chosen antibody (against CDK6, LC3B, ubiquitin, AMBRA1, or CUL4A) or negative control IgG for 1–2 h at room temperature. Then, the antigen/antibody complex was bound to protein A/G magnetic beads overnight with rotation at 4 °C. After washing with IP lysis buffer and purified water, the antigen/antibody complex bound to A/G magnetic beads was collected in elution buffer and subjected to western blot analysis.

### Flow Cytometric Analysis

For cell cycle analysis, cells were trypsinized, washed with cold PBS, and fixed in 70% ice‐cold ethanol at −20 °C overnight. Then, the cells were washed with PBS and incubated with 10 mg mL^−1^ RNase A (Qiagen, Germany) and 400 mg mL^−1^ propidium iodide (Keygen, China) at 4 °C for 30 min. For apoptosis analysis, cells were suspended in binding buffer with PE‐conjugated Annexin‐V (eBioscience, USA). After 30 min of incubation at 4 °C, a FACScan flow cytometer (BD Biosciences, USA) was used to measure the fluorescence intensity. Modfit LT 3.2 and FlowJo 7.6.1 software were used to calculate the cell cycle distribution profiles and the percentage of apoptotic cells.

### Immunohistochemistry (IHC) and Immunofluorescence (IF)

IHC assays were conducted according to standard protocols on 4 mm paraffin sections of tissue samples embedded in paraffin. Tumor sections were dewaxed, rehydrated, and incubated with primary antibodies against LC3B (1:200), CDK6 (1:200), Cyclin D1 (1:200), AMBRA1 (1:200), LAMP1 (1:200), p62 (1:200), and Beclin1 (1:200) overnight at 4 °C. The sections were subsequently incubated with secondary antibodies, and diaminobenzidine tetrahydrochloride (DAB, ZSBIO, China) was used to detect HRP activity. Images were taken with a Leica DM2500 microscope (Leica, Germany). After the indicated treatment, the cells were fixed in 4% paraformaldehyde and permeabilized with 0.5% Triton X‐100 in PBS. After blocking with goat serum, the cells were stained using primary antibodies against LAMP1 (1:200), p62 (1:200), TFEB (1:200), and TFE3 (1:200) and secondary antibodies conjugated with Alexa Fluor. After the cells were incubated with 4′,6‐diamidino‐2‐phenylindole (DAPI), the fluorescence signals were visualized using a fluorescence microscope. To detect autophagic flux, cells were infected with mRFP‐GFP‐LC3 virus (Hanbio Biotechnology Co., Ltd, Shanghai, China) and stained with Lysosome‐Tracker Red (Solarbio, China). Images were acquired using a confocal microscope (Carl Zeiss, Germany).

### Transmission Electron Microscopy (TEM)

SCLC cells were fixed in 2.5% glutaraldehyde containing 0.1 mol L^−1^ sodium cacodylate and transferred to cacodylate buffer with 0.1 m sucrose to stop fixation. After being washed in cacodylate buffer, the cells were embedded in an epoxy resin/propylene oxide 1:1 mixture. Ultrathin sections were then cut on a microtome, placed on copper grids, stained with uranyl acetate and lead citrate, and observed under a transmission electron microscope (JEOL, USA).

### Luciferase Assay

The E2F1 promoter (3601 bp relative to the ATG site), CTSB promoter (1201 bp relative to the ATG site) and LAMP1 promoter (3801 bp relative to the ATG site) were cloned into the luciferase reporter gene vector pGL4.23 (Promega, USA). H69, H69AR, and A549 cells were transfected with E2F1‐, CTSB‐, and LAMP1‐responsive luciferase reporters containing the above promoter elements in combination with pTK‐RL (a kinase promoter upstream of Renilla luciferase). After treatment, the cells were lysed, and luciferase activity was detected using Dual Reporter Assay kits (Promega, USA). Relative light units were calculated as firefly and Renilla luciferase ratio values.

### Small Cell Lung Cancer Mouse Models

Rb1^flox/flox^, Trp53^flox/flox^, and Pten^flox/flox^ (RTP) mice were provided by Hefei Cancer Hospital, Chinese Academy of Sciences, and Rb1^flox/flox^, Trp53^flox/flox^, and Myc^LSL/LSL^ (RPM) mice were obtained from the Jackson Laboratory (#02 9971). Mice aged six to eight weeks were used for experiments. To generate tumors in the lungs of RTP and RPM mice, replication‐deficient adenoviruses expressing Cre‐recombinase (Ad‐Cre) were delivered to the lungs by intratracheal installation. Mice were treated with one dose of 1 × 10^8^ PFU of Ad‐CMV‐Cre (ShanDong WeiZhen Biosciences Company). RPM mice were analyzed for tumor formation and progression at 3–6 weeks post‐adenoviral infection while RTP mice were analyzed at 4–6 months post‐infection. For treatment studies, mice were evaluated by MRI (Bruker, 7.0T) or in vivo imaging (PerkinElmer, IVIS Spectrum) to quantify lung tumor burden before randomization and after drug treatment for efficacy evaluation.

### Tumor Xenograft Formation in Mice

Animal studies were approved by the animal ethics committee of the Southern Medical University of China. Female BALB/c nude mice aged 3–4 weeks were purchased from the Experimental Animal Center of Southern Medical University. A total of 1 × 10^7^ SCLC cells were suspended in 100 µL PBS per female nude mouse. Tumors were allowed to grow for 7–10 days. When tumors reached an average size of 100–150 mm^3^, the BALB/c nude mice were randomly divided into four groups. For the group receiving chemotherapy, mice implanted with tumor cells were treated weekly with cycles of cisplatin (Hausen Pharmaceutical, China; 2.5 mg kg^−1^, i.p. injection on day 1) and etoposide (Vepesid, Australia; 4 mg kg^−1^, i.p. injection on days 1–3). For the group receiving CDK4/6 inhibitors, palbociclib (MedChemExpress, USA; 100 mg kg^−1^, administered on days 1–5, weekly) was given orally. Three weeks later, these mice were euthanized, and the tumors were obtained. Tumor sizes were measured with electronic calipers every 3–4 days and tumor volume was calculated using (length × width^2^)/2.

### SCLC Patient‐Derived Xenograft (PDX) Model

Fresh human tumor tissues from consenting patients with SCLC were collected at Guangdong Provincial People's Hospital and the Zhujiang Hospital of Southern Medical University with the approval of the Institutional Review Board. All animal studies were conducted in accordance with the Institutional Animal Care and Use Committee (IACUC)‐approved animal protocols of Southern Medical University. The diagnosis of SCLC was confirmed by a pathologist. Primary surgical tumor samples or metastatic lymph node resection samples of SCLC were cut into 3–5 mm^3^ fragments and transplanted into the severely immunocompromised B‐NDG mice (BIOCYTOGEN, China) under the endothelium 6 h after surgical resection. After 2 months, when the tumor size exceeded 1000–1500 mm^3^, xenograft fragments were immediately implanted into new B‐NDG mice for passaging. The PDX model with chemotherapy resistance received chemotherapy drugs for at least 6 cycles during each generation. In each cycle, mice implanted with tumors were treated every 10 days with cisplatin (2.5 mg kg^−1^, i.p. injection on day 1) and etoposide (4 mg kg^−1^, i.p. injection on days 1–3). The chemotherapy‐sensitive and chemotherapy resistant PDX models were treated with CDK4/6 inhibitors in the same way as the BALB/c nude mice with tumor xenografts.

### Statistical Analysis

All data in the text and figures were analyzed using GraphPad Prism 8.0 (GraphPad Software, USA) and are presented as the mean ± SDs. Statistical comparisons of data were performed using Student's *t* test or one‐way ANOVA and the differences were considered statistically significant when the *p* value was < 0.05. All experiments have been repeated at least three times, with a biological population of 6 or more. A chi‐square test was used to analyze relationships between CDK6 expression and clinicopathological features. OS curves were analyzed using Kaplan–Meier method and were compared among groups using a log‐rank test. Univariate and multivariate analyses were performed using Cox regression analysis. *p* < 0.05 was considered statistically significant.

### Ethics Approval

The animal study was approved by the Animal Care and Use Committee of Southern Medical University (LAEC‐2020‐129; Guangzhou, China). All methods were carried out in accordance with relevant guidelines and regulations, the study was carried out in compliance with the ARRIVE guidelines. The patient study was approved by the Patient Samples Committee of Fujiang Province (K2017‐12‐024).

### Statement of Translational Relevance

The formation of resistance to chemotherapy drugs has been a major problem in SCLC therapy. Some new targets should be considered proteinal actionable targets to overcoming the challenge. In this study, the function and underlying mechanism of CDK4/6 signaling pathway in SCLC chemoresistance were uncovered. Moreover, AMBRA1 was identified as an important downstream mediator in SCLC chemotherapy resistance. These results showed that CDK6 protein expression was associated with poor chemotherapy response, these results suggested that CDK6 protein expression could serve as a valuable predictive factor for chemoresistance of SCLC. Otherwhile, some of these results suggested that CDK4/6 inhibitors combined with chemotherapeutic drugs exerted a strong tumor‐inhibitory effect in SCLC mouse tumors and chemoresistance SCLC cells. This study suggested that CDK4/6 inhibitors might serve as potential therapeutic options for SCLC patients.

## Conflict of Interest

The authors declare no conflict of interest.

## Author Contributions

Y.W., X.S., L.Z., S.L., and D.L. contributed equally to this work. Y.W., X.S., L.Z., S.L., D.L., X.C., X.S., F.Z., Z.W., and Q.Z. performed the experiments. Y.W., X.S., L.D., and L.G. designed the study, analyzed, and interpreted the results, wrote, and edited the manuscript. Y.W., L.Z., S.L., D.L., X.C., X.S., F.Z., and Q.Z. assisted with main experiments. L.D. and L.G. provided essential reagents and techniques for this study and reviewed the manuscript. Y.W., S.X., L.Z., S.L., D.L., X.C., X.S., F.Z., Q.Z., L.D., and L.G. approved the final version of manuscript. L.G. conceived and supervised the study.

## Supporting information

Supporting Information

## Data Availability

The data that support the findings of this study are available in the supplementary material of this article.
