# Supplementary material for: CDK4/6 Inhibitors Impede Chemoresistance and Inhibit Tumor Growth of Small Cell Lung Cancer
Source: Adv Sci (Weinh). 2024 Aug 13;11(38):2400666. doi: 10.1002/advs.202400666 (PMC11481398; doi:10.1002/advs.202400666)
Supplement: Supplementary file 1 — Supporting Information [file ADVS-11-2400666-s001.pdf]

ADVANCED SCIENCE
Open Access

Supporting Information

for *Adv. Sci.*, DOI 10.1002/advs.202400666

CDK4/6 Inhibitors Impede Chemoresistance and Inhibit Tumor Growth of Small Cell Lung Cancer

Yang Wen, Xue Sun, Lingge Zeng, Shumei Liang, Deyu Li, Xiangtian Chen, Fanrui Zeng, Chao Zhang, Qiongyao Wang, Qinsong Zhong, Ling Deng and Linlang Guo**

Supplementary

Figure S1

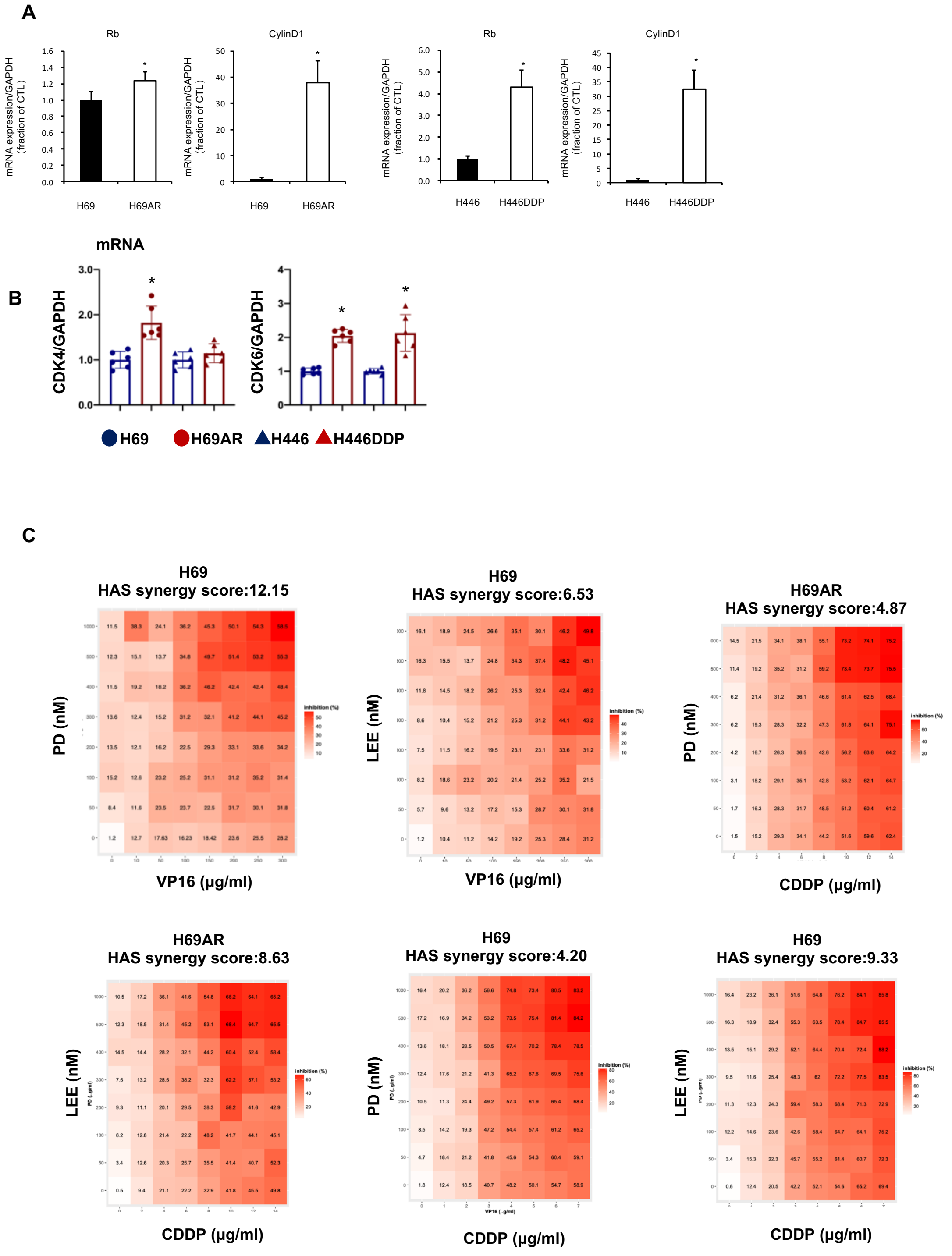

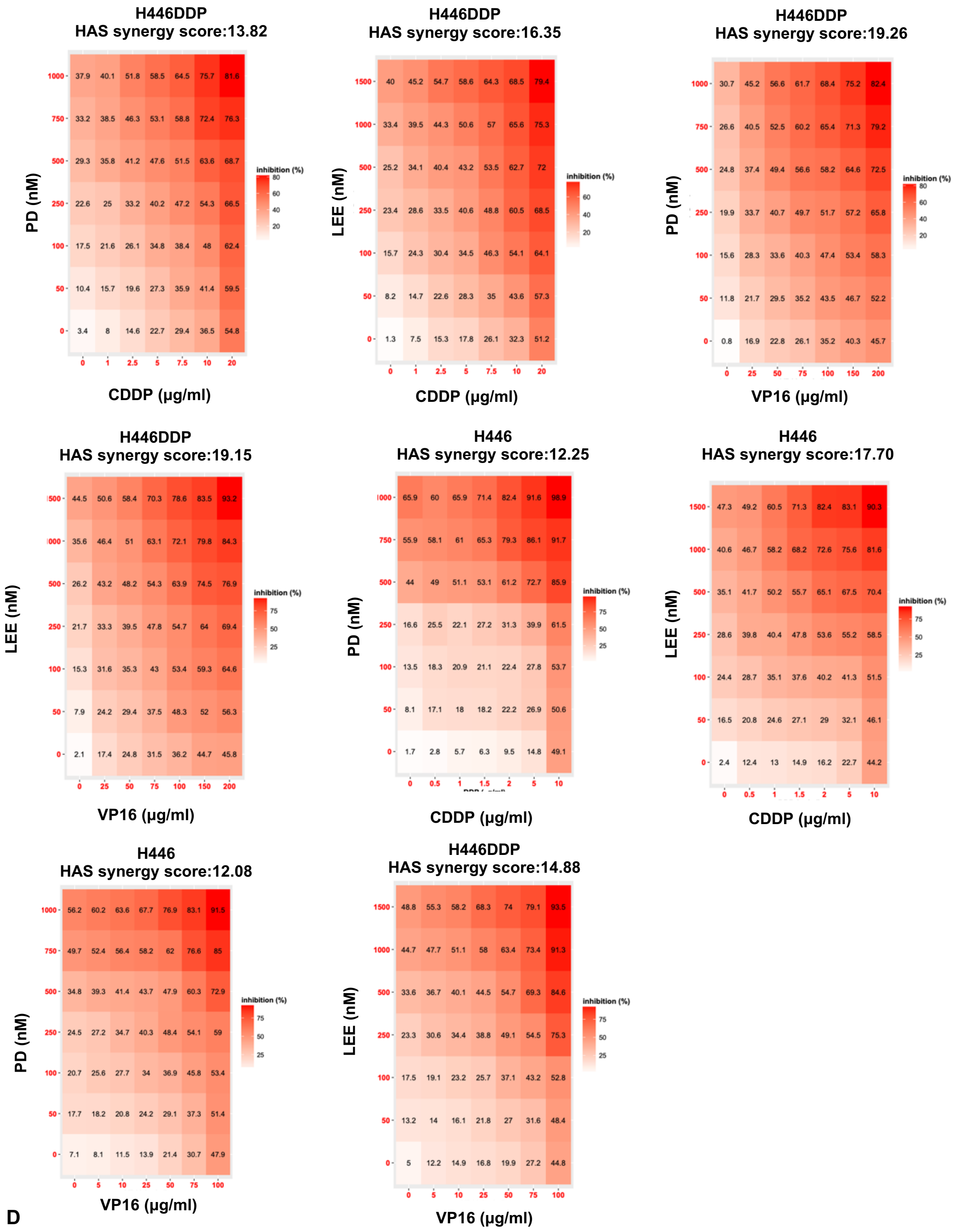

D

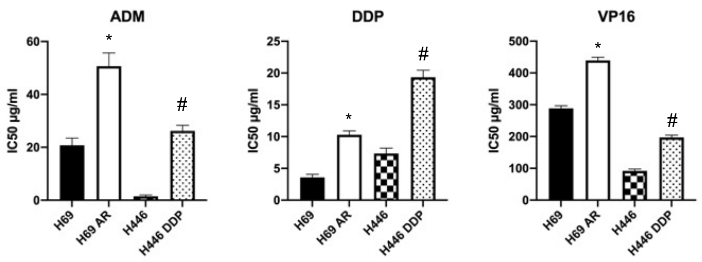

Figure S1. (A) mRNA levels of RB1 and CyclinD1 in H69, H69AR, H446 and H446DDP cells (n = 6). (B) CDK4 and CDK6 mRNA and protein expression

levels in H69, H69AR, H446 and H446DDP cells (n = 6). (C) The synergy effect of PD and LEE with VP16 and CDDP of cell viability inhibition in H69, H446 and H446DDP. (D) IC50 values of H69, H69AR, H446 and H446DDP cells treated with ADM, DDP and VP16 (n = 6). The data are shown as the mean ± SD, $*p<0.05$. For panel D, $*p<0.05$ and # $p<0.05$ indicating different between H69-H69AR and H446-H446DDP respectively.

Figure S2

A

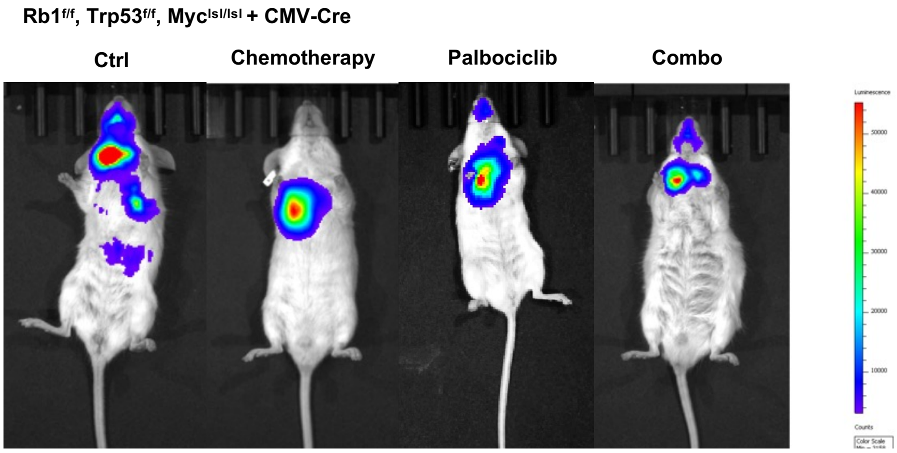

B

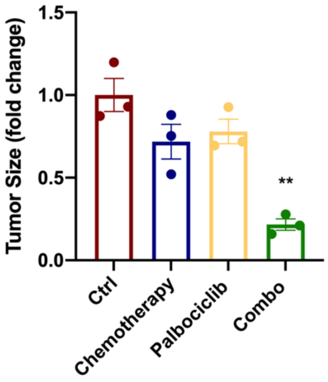

C

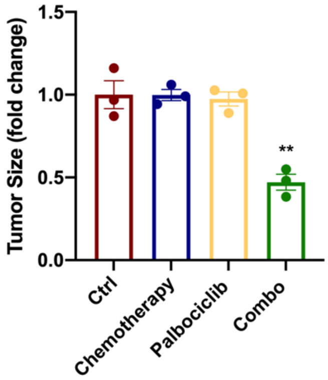

D

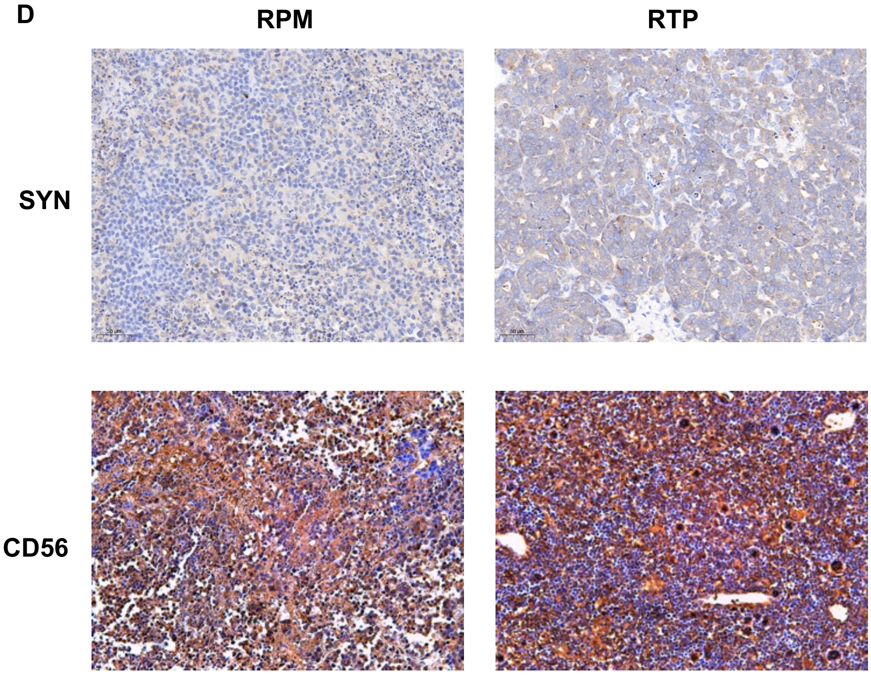

Figure S2. (A) Live image of tumors in RPM mice' lungs. (B) Statistical map of the tumor area in RPM mice (n = 3). (C) Statistical map of the tumor area in RTP mice (n = 3). (D) CD56 and SYN staining in tumors from RPM and RTP mice. CD56 and SYN are neuroendocrine markers. The data are shown as the mean ± SD, $^{**}p<0.01$.

Figure S3

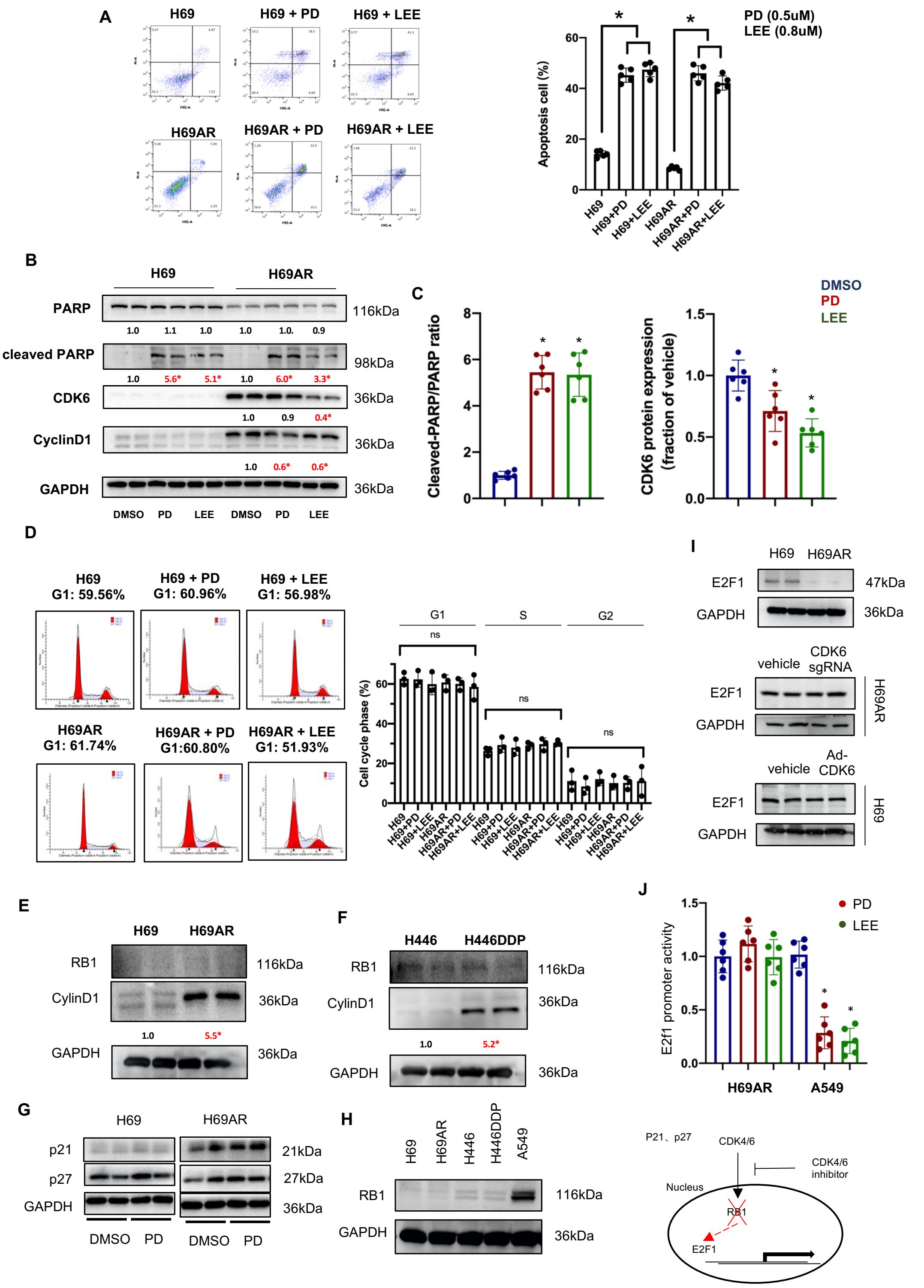

Figure S3. Cell cycle modulation is not responsible for the ability of CDK4/6 inhibitors to improve chemosensitivity and reverse chemoresistance in SCLC. (A) Annexin V-FITC/PI levels in H69 and H69AR cells treated with PD or LEE detected by flow cytometry. The statistical map is shown in the right panel (n = 6). (B and C) The protein expression levels of PARP, cleaved PARP, CDK6,

Cyclin D1 and GAPDH were detected in H69 and H69AR cells with or without PD and LEE treatment. (n = 6). (D) The cell cycle distribution in H69 and H69AR cells with or without PD and LEE treatment was analyzed via flow cytometry and the statistical map is shown in the right panel (n = 6). (E and F) Protein expression levels of RB1 and Cyclin D1 in H69, H69AR, H446 and H446DDP cells (n = 6). (G) Protein expression levels of p21 and p27 in H69 and H69AR cells with or without PD treatment (n = 6). (H) RB1 expression in SCLC cell lines (H69, H69AR, H446 and H446DDP) and a lung adenocarcinoma cell line (A549) (n = 6). (I) E2F1 protein expression in H69 cells, H69AR cells, CDK6 sgRNA-transfected H69AR cells and CDK6-overexpressing adenovirus-infected H69 cells (n = 6). (J) The viability of the E2F1 transcription factor in PD- and LEE-treated H69AR and A549 cells was detected using a luciferase assay (n = 6). The data are shown as the mean ± SD, $^{*}p<0.05$; ns, no signicicant.

Figure S4

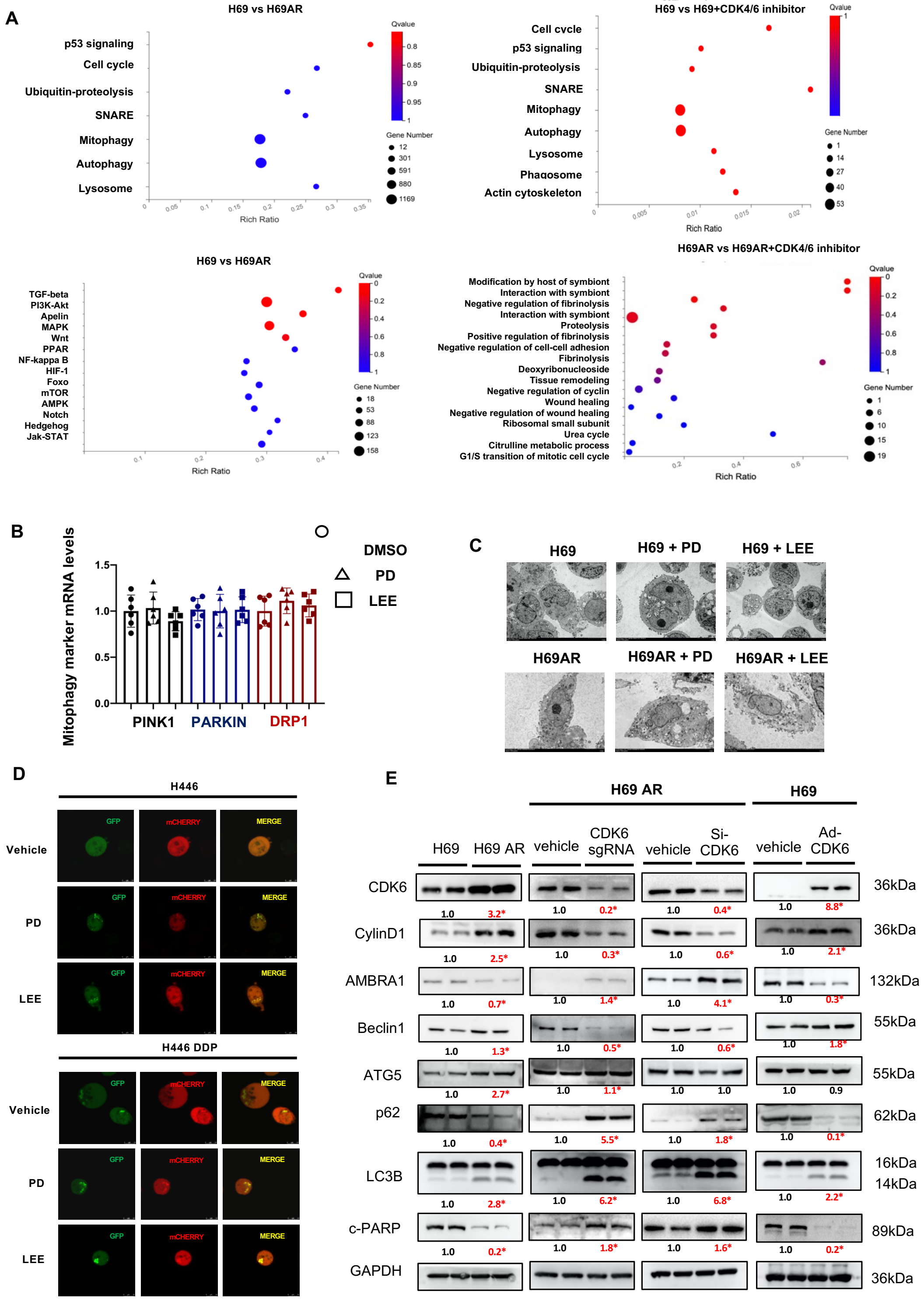
A
H69 vs H69AR
p53 signaling
Cell cycle
Ubiquitin-proteolysis
SNARE
Mitophagy
Autophagy
Lysosome
Rich Ratio
Qvalue
Gene Number
H69 vs H69+CDK4/6 inhibitor
Cell cvcle
p53 signaling
Ubiquitin-proteolysis
SNARE
Mitophagy
Autophagy
Lysosome
Phagosome
Actin cytoskeleton
H69 vs H69AR
TGF-beta
PI3K-Akt
Apelin
MAPK
Wnt
PPAR
NF-kappa B
HIF-1
Foxo
mTOR
AMPK
Notch
Hedgehog
Jak-STAT
H69AR vs H69AR+CDK4/6 inhibitor
Modification by host of symbiont
Interaction with symbiont
Negative regulation of fibrinolysis
Interaction with symbiont
Proteolysis
Positive regulation of fibrinolysis
Negative regulation of cell-cell adhesion
Fibrinolysis
Deoxyribonucleoside
Tissue remodeling
Negative regulation of cyclin
Wound healing
Negative regulation of wound healing
Ribosomal small subunit
Urea cycle
Citrulline metabolic process
G1/S transition of mitotic cell cycle
B
Mitophagy marker mRNA levels
PINK1
PARKIN
DRP1
DMSO
PD
LEE
C
H69
H69 + PD
H69 + LEE
H69AR
H69AR + PD
H69AR + LEE
D
H446
H446 DDP
Vehicle
PD
LEE
GFP
mCHERRY
MERGE
E
H69 AR
H69
H69
H69 AR
vehicle
CDK6 sgRNA
vehicle
Si-CDK6
vehicle
Ad-CDK6
CDK6
CylinD1
AMBRA1
Beclin1
ATG5
p62
LC3B
c-PARP
GAPDH
36kDa
36kDa
132kDa
55kDa
55kDa
62kDa
16kDa
14kDa
89kDa
36kDa

F

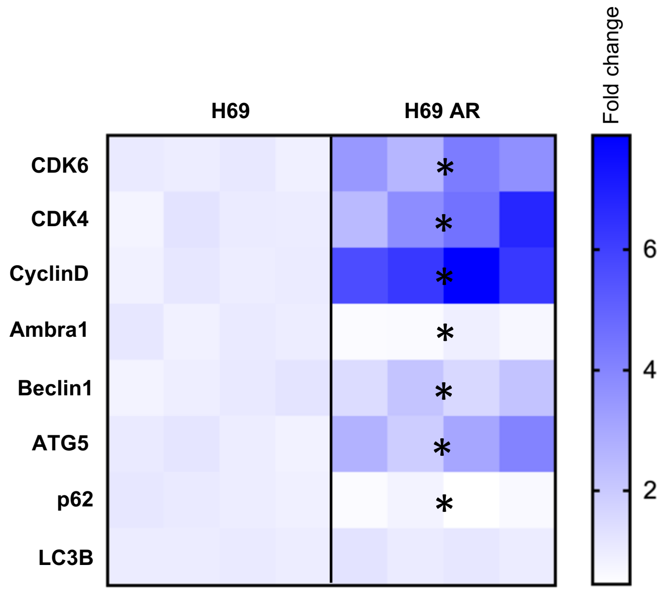

G

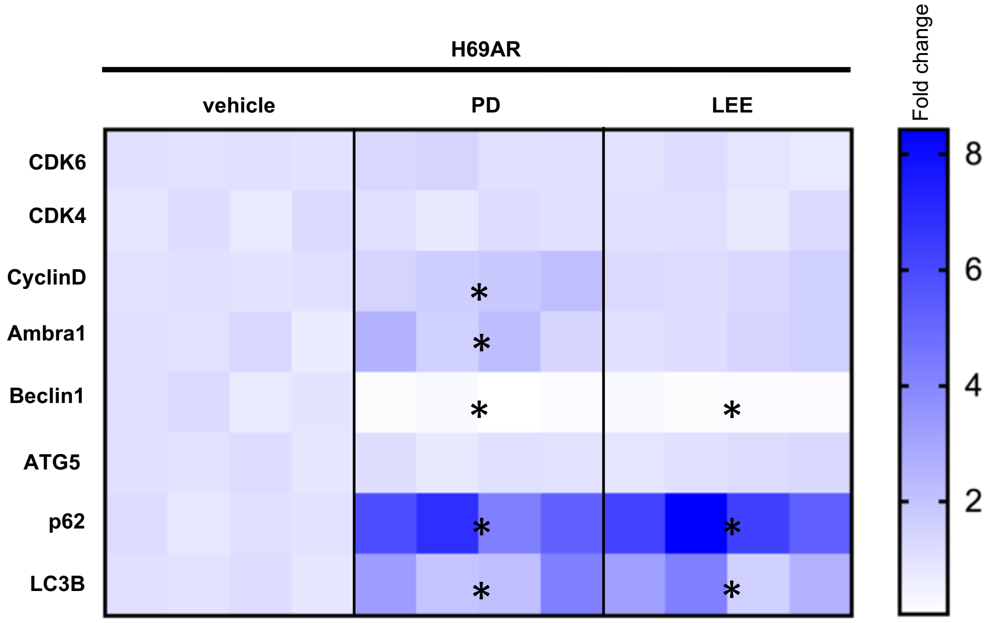

Figure S4. (A) After RNA sequencing of H69, H69AR, H69 cells treated with CDK4/6 inhibitors and H69AR cells treated with CDK4/6 inhibitors, KEGG analysis of the differentially expressed genes was performed (the pathway which $p<0.05$ were shown). (B) mRNA levels of PINK1, PARKIN and DRP1 in PD- and LEE-treated H69AR cells (n = 6). (C) Transmission electron microscopy images of H69 and H69AR cells treated with or without PD and LEE (low magnification). Scale bar: 5 μm. (D) Autophagic flux was analyzed in H446 and H446DDP cells treated with or without PD and LEE after transfection

with the mRFP-GFP-LC3 dual reporter virus. Scale bar: 10 μm. (E) Cell cycle-related proteins (CDK6 and Cyclin D1) and autophagy-related proteins (AMBRA1, Beclin1, ATG5, p62, LC3B and c-PARP) were detected in H69 cells, H69AR cells, CDK6 sgRNA-transfected H69AR cells, CDK6 siRNA-transfected H69AR cells and CDK6-overexpressing adenovirus-infected H69 cells (n = 6). (F) mRNA levels of CDK4, CDK6, CyclinD, Ambra1, Beclin1, ATG5, p62 and LC3B in H69 and H69AR cells were shown (n = 6). (G) mRNA levels of CDK4, CDK6, CyclinD, Ambra1, Beclin1, ATG5, p62 and LC3B in H69 cells treated with PD (0.5μM) and LEE (0.8μM) were shown (n =6). The data are shown as the mean ± SD, $^*p<0.05$.

Figure S5

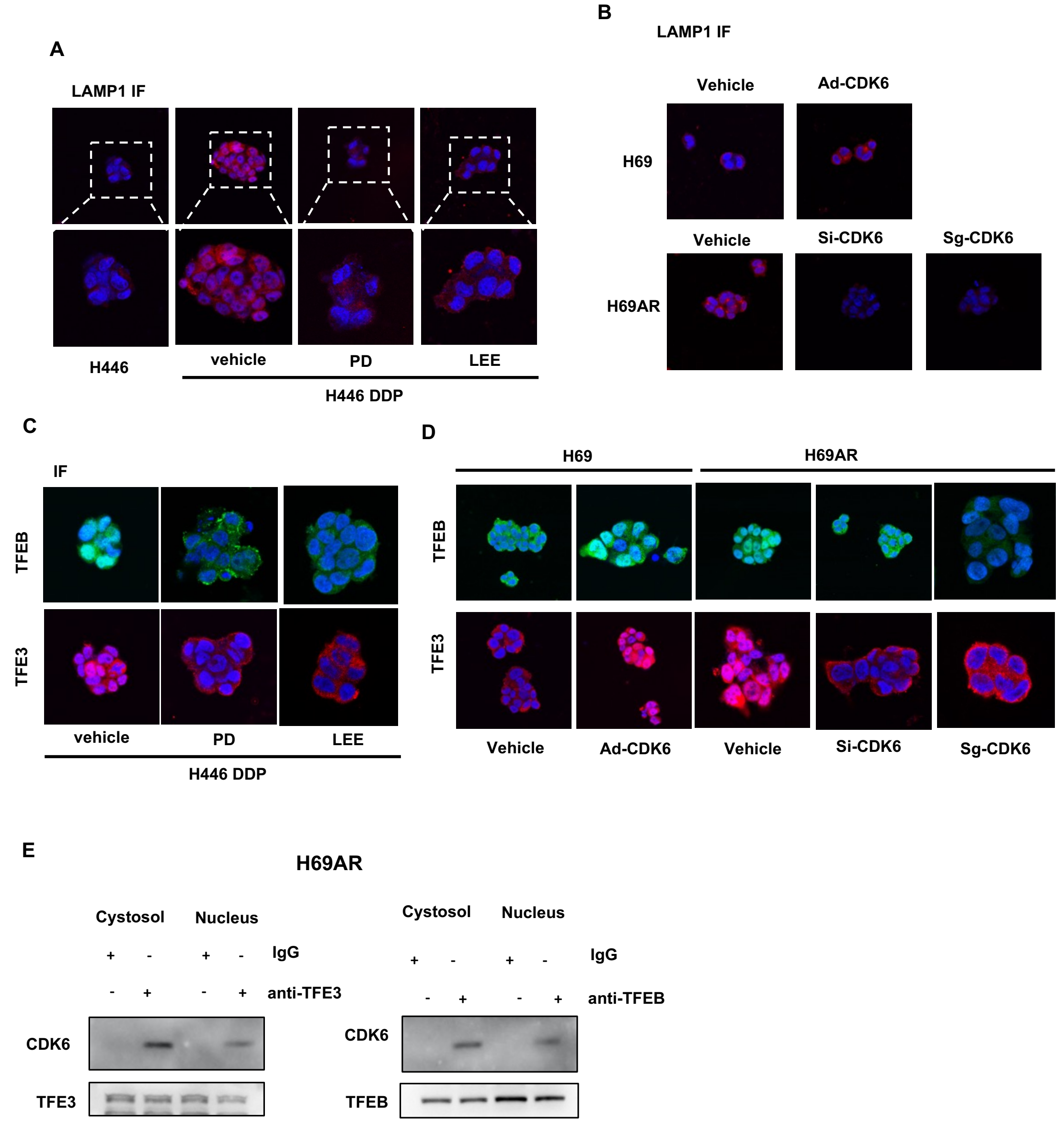

Figure S5. (A) LAMP1 immunofluorescence images of H446 and H446DDP cells treated with PD and LEE. (B) LAMP1 immunofluorescence images of H69 cells and Ad-CDK6-transfected H69 cells (left panel) and H69AR cells and H69AR cells transfected with si-CDK6 and sg-CDK6 (right panel). (C) Immunofluorescence images showing TFEB and TFE3 expression in H69 cells. (D) Immunofluorescence images showing TFEB and TFE3 expression in Ad-CDK6-transfected H69 cells (left panel) and H69AR cells and H69AR cells transfected with si-CDK6 and sg-CDK6 (right panel). Scale bar: 10 μm. (E) Immunoprecipitation of CDK6 with TFEB and TFE3 in the nucleus and cytoplasm of H69AR cells.

Figure S6

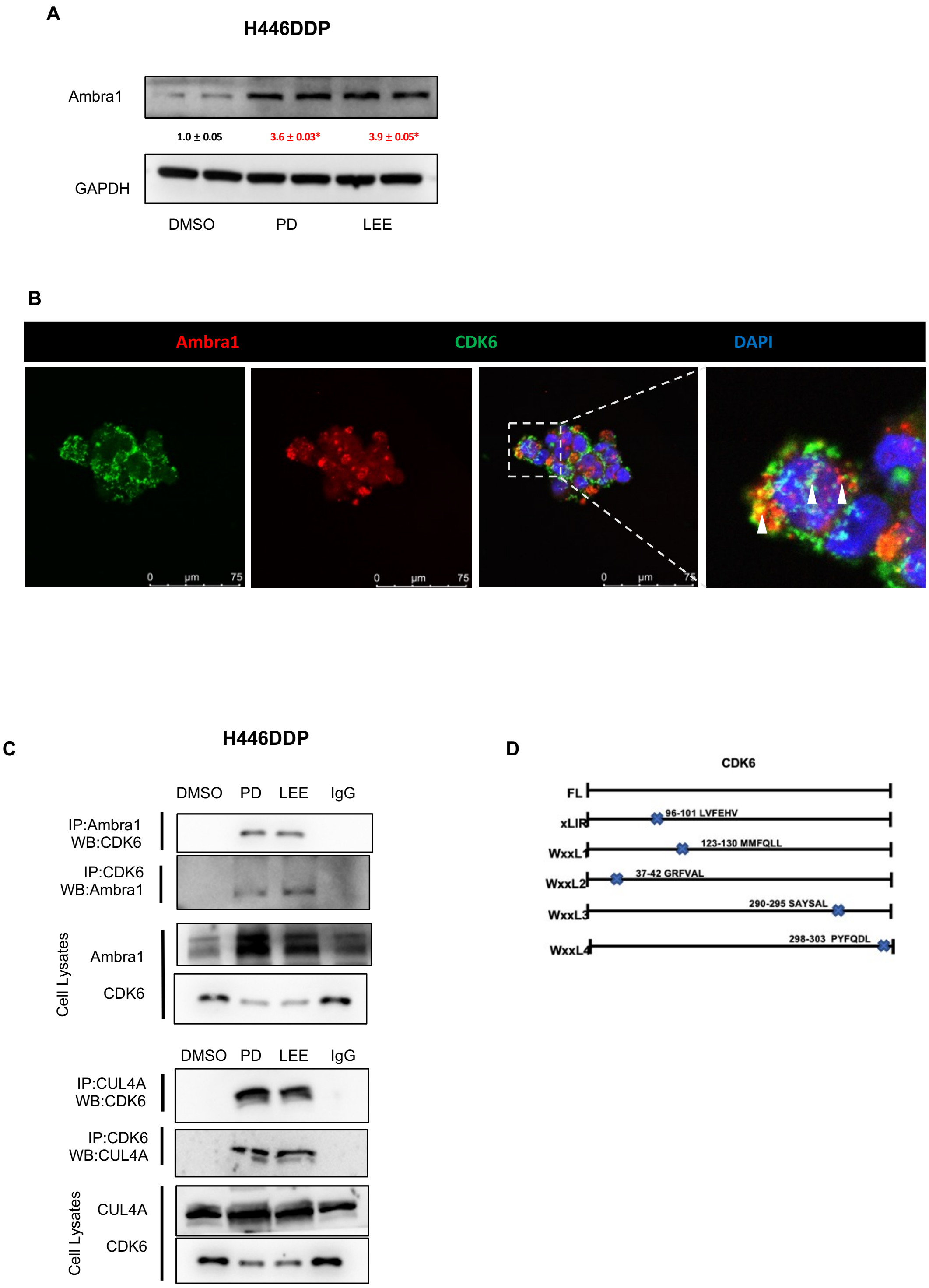

Figure S6. (A) AMBRA1 protein expression in H446DDP cells treated with PD and LEE. (B) Immunofluorescence images showing the overlay of AMBRA1 and CDK6 in H69AR cells. Scale bar: 10 μm. (C) Immunoprecipitation of CDK6 with AMBRA1 and CUL4A in PD- and LEE-treated H446DDP cells. (D) iLIR

analysis using the iLIR database revealed the amino acid sites in the CDK6 protein most likely to bind LC3B.

Figure S7

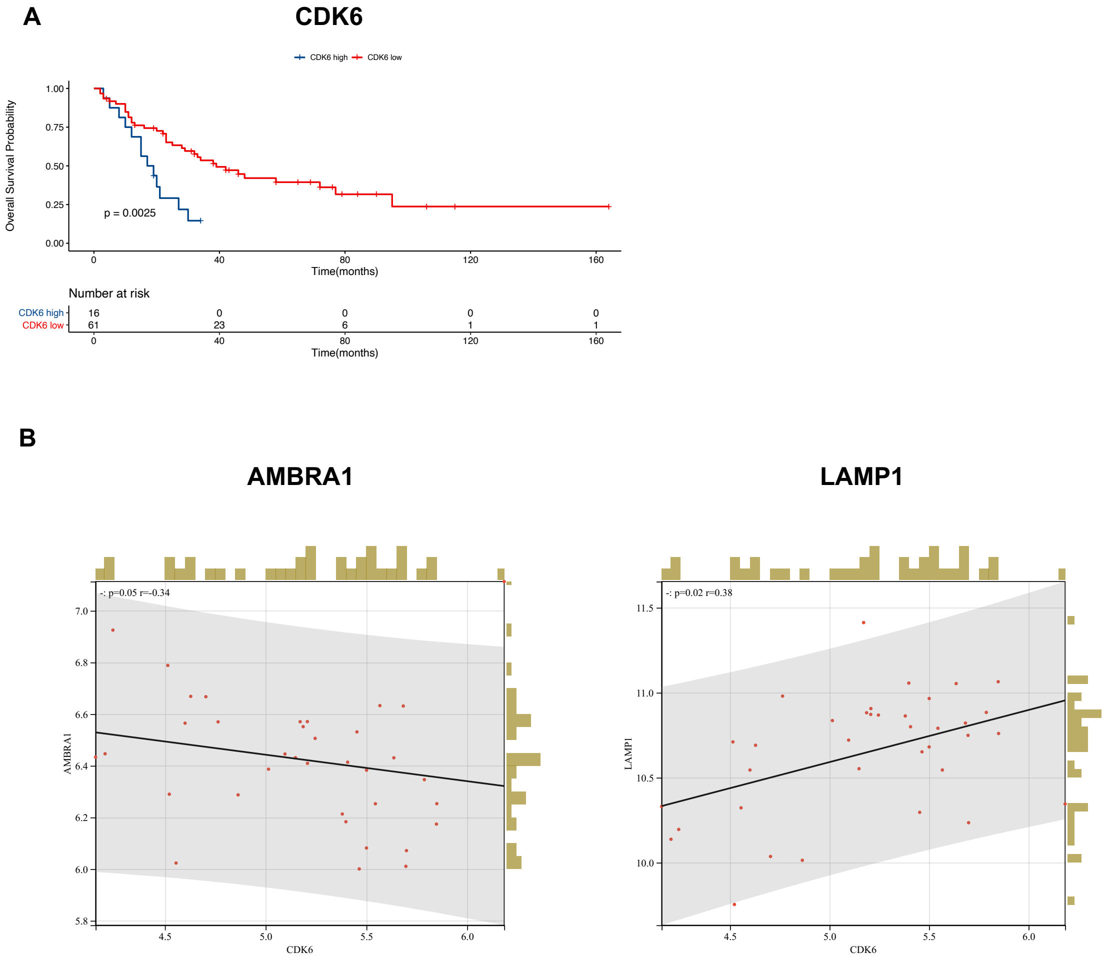

Figure S7. (A) Kaplan-Meier survival curve showing the correlation between CDK6 expression and overall survival in EGAS00001000925. (B) Pearson correlation coefficient results of for CDK6-AMBRA1 and CDK6-LAMP1 in GSE149507.
